# Supplementary material for: Smokers’ strategies across social grades to minimise the cost of smoking in a period with annual tax increases: evidence from a national survey in England
Source: BMJ Open. 2019 Jun 25;9(6):e026320. doi: 10.1136/bmjopen-2018-026320 (PMC6597620; doi:10.1136/bmjopen-2018-026320)
Supplement: Supplementary data [file bmjopen-2018-026320supp001.pdf]

**SUPPLEMENTARY FILE**

Mirte AG Kuipers, Timea R Partos, Ann McNeill, Emma Beard, Anna Gilmore, Robert West, Jamie Brown
Smokers' strategies across social grades to minimise the cost of smoking in a period with annual tax increases: Evidence from a national survey in England
BMJ Open

**Supplementary Table 1:** Results from weighted Generalised Additive Models (GAM) for cost of smoking, with total cigarette consumption and proportion of RYO use.

| | **Percentage difference in cost of smoking (100β with 95%CI)** |
|---|---|
| | **Model 6**<br>Adjusted for other cost-minimising strategies |
| Age (per 10 years increase) | -0.29 (-0.85 to 0.27) |
| Gender | |
| Male | ref |
| Female | 1.29 (-0.51 to 3.08) |
| Social grade | |
| Low | ref |
| Middle | 2.35 (0.40 to 4.30) |
| High | -1.24 (-4.06 to 1.57) |
| Total cigarette consumption (per 10 cigarettes decrease) | -10.0 (-9.83 to -10.17) |
| Proportion of RYO cigarette consumption[a] | -70.41 (-72.29 to -68.53) |
| Use of illicit sources | -9.91 (-13.21 to -6.62) |
| Use of cross-border sources | -8.68 (-11.93 to -5.42) |

[a] Represents the difference between 0% RYO and 100% RYO cigarettes of total consumption.

**Supplementary Table 2:** Results from weighted Generalised Additive Models (GAM) for price per cigarette.

| | **Percentage difference in price per cigarette (100β with 95%CI)** |
|---|---|
| | **Model 6**<br>Adjusted for other cost-minimising strategies |
| Age (per 10 years increase) | -0.59 (-1.06 to -0.11) |
| Gender | |
| Male | ref |
| Female | 1.70 (0.18 to 3.23) |
| Social grade | |
| Low | ref |
| Middle | 5.41 (3.75 to 7.07) |
| High | 9.52 (7.12 to 11.91) |
| Factory-made cigarette consumption (per 10 cigarettes decrease) | 0.81 (0.97 to 0.65) |
| RYO cigarette consumption (per 10 cigarettes decrease) | 7.76 (7.93 to 7.59) |
| Use of illicit sources | -12.10 (-14.90 to -9.29) |
| Use of cross-border sources | -11.17 (-13.94 to -8.40) |

**Supplementary Table 3:** Results from weighted Generalised Additive Models (GAM) for cost of smoking, in the population from which respondents were not excluded based on the reported value for price/cigarette (N=17,789).

| | **Percentage difference in cost of smoking (100β with 95%CI)** |
|---|---|
| | **Model 6**<br>Adjusted for other cost-minimising strategies |
| Age (per 10 years increase) | 0.39 (-0.25 to 1.02) |
| Gender | |
| Male | ref |
| Female | 1.48 (-0.57 to 3.52) |
| Social grade | |
| Low | ref |
| Middle | 0.32 (-1.90 to 2.54) |
| High | -3.16 (-6.44 to 0.12) |
| Factory-made cigarette consumption[a] | |
| Per 10 cigarettes decrease | -12.27 (-12.48 to -12.05) |
| RYO cigarette consumption[b] | |
| Per 10 cigarettes decrease | -4.56 (-4.77 to -4.34) |
| Use of illicit sources[c] | |
| No use of illicit sources in last 6 months | ref |
| Used illicit sources in last 6 months | -9.12 (-12.83 to -5.42) |
| Cross-border purchase[d] | |
| No cross-border purchase in last 6 months | ref |
| Cross-border purchase in last 6 months | -14.87 (-18.57 to -11.18) |

RYO = roll-your-own

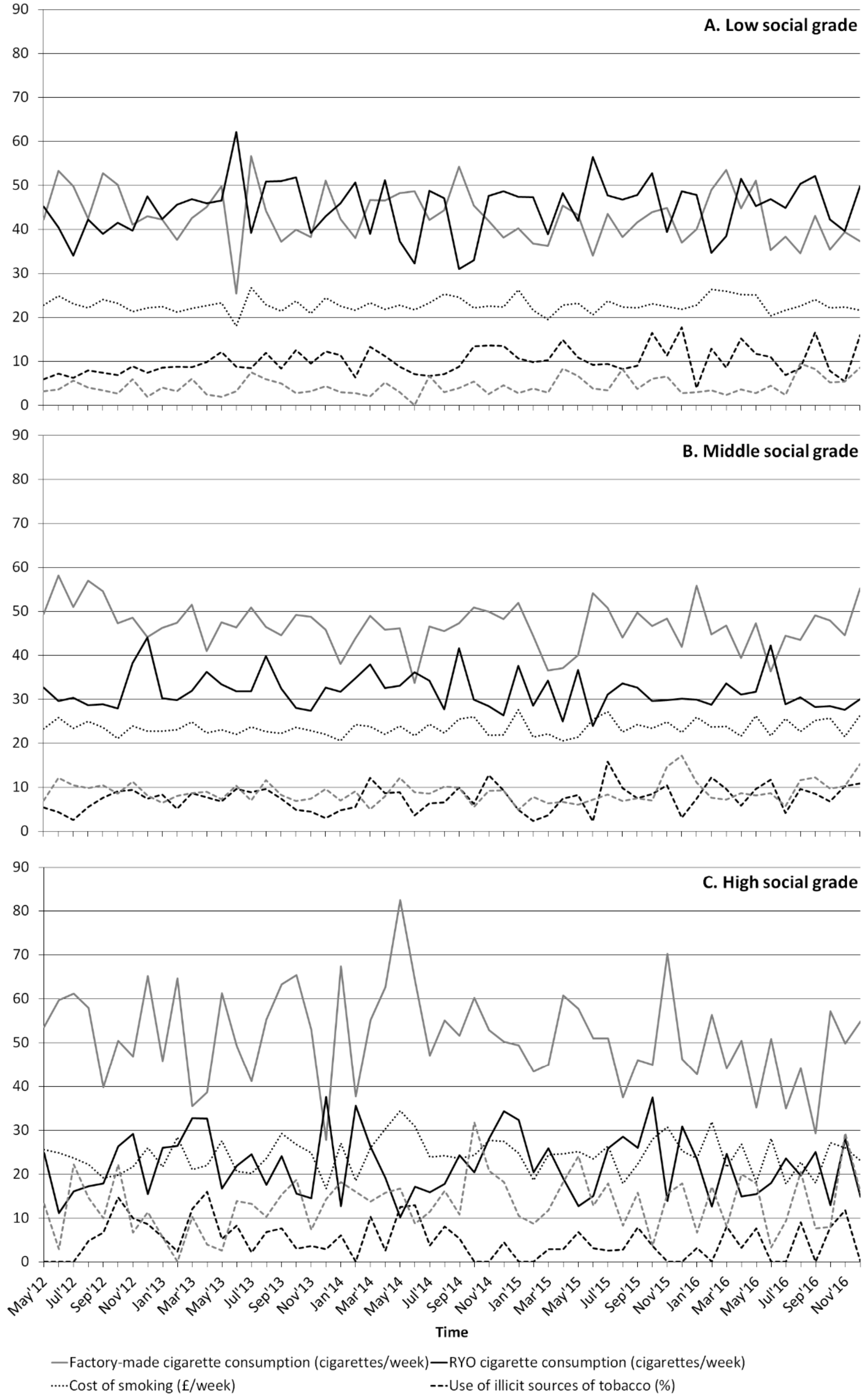


**Supplementary Figure 1:** Weighted trends in cost of smoking, cigarette consumption, use of illicit sources, and cross-border purchase among smokers in England, stratified by social grade.
