# Supplementary material for: Smokers’ strategies across social grades to minimise the cost of smoking in a period with annual tax increases: evidence from a national survey in England
Source: BMJ Open. 2019 Jun 25;9(6):e026320. doi: 10.1136/bmjopen-2018-026320 (PMC6597620; doi:10.1136/bmjopen-2018-026320)
Supplement: Author's manuscript [file bmjopen-2018-026320.draft_revisions.pdf]

BMJ Open is committed to open peer review. As part of this commitment we make the peer review history of every article we publish publicly available.

When an article is published we post the peer reviewers' comments and the authors' responses online. We also post the versions of the paper that were used during peer review. These are the versions that the peer review comments apply to.

The versions of the paper that follow are the versions that were submitted during the peer review process. They are not the versions of record or the final published versions. They should not be cited or distributed as the published version of this manuscript.

BMJ Open is an open access journal and the full, final, typeset and author-corrected version of record of the manuscript is available on our site with no access controls, subscription charges or pay-per-view fees (http://bmjopen.bmj.com).

If you have any questions on BMJ Open's open peer review process please email info.bmjopen@bmj.com

BMJ Open

Smokers' strategies across social grades to minimise the cost of smoking in a period with annual tax increases: Evidence from a national survey in England

Journal:	*BMJ Open*
Manuscript ID	bmjopen-2018-026320
Article Type:	Research
Date Submitted by the Author:	27-Aug-2018
Complete List of Authors:	Kuipers, Mirte; Academic Medical Center, University of Amsterdam,<br>Partos, Timea; Kings College London, Addictions<br>Brown, Jamie; University College London, Psychology & Language Sciences<br>Beard, Emma; UCL,<br>Gilmore, Anna; Tobacco Control Research Group, UK Centre for Tobacco and Alcohol Studies, Department for Health, University of Bath<br>West, Robert; University College London<br>McNeill, Ann; King's College London, UK Centre for Tobacco Control Studies, National Addiction Centre, Institute of Psychiatry
Keywords:	EPIDEMIOLOGY, PREVENTIVE MEDICINE, PUBLIC HEALTH

SCHOLARONE™
Manuscripts

For peer review only

Smokers' strategies across social grades to minimise the cost of smoking in a period with annual tax increases: Evidence from a national survey in England

Mirte AG Kuipers, PhD, corresponding author
Department of Epidemiology and Public Health, Health Behaviour Research Centre, University College London, London, UK
Department of Public Health, Amsterdam Public Health Research Institute, Academic Medical Center – University of Amsterdam, P.O. Box 22660, 1100 DD, Amsterdam, The Netherlands
E-mail address: m.a.kuipers@amc.uva.nl

Timea R Partos, PhD
Department of Addictions, Institute of Psychiatry, Psychology and Neuroscience, King's College London, 4 Windsor Walk, Denmark Hill, London, SE5 8BB, UK

Jamie Brown, PhD
Research Department of Clinical, Educational and Health Psychology, University College London, 1-19 Torrington Place, London, WC1E 7HB, UK

Emma Beard, PhD
Research Department of Clinical, Educational and Health Psychology, University College London, 1-19 Torrington Place, London, WC1E 7HB, UK

Anna Gilmore, PhD
Tobacco Control Research Group, Department for Health, University of Bath, Claverton Down, Bath, BA2 7AY, UK

Robert West, PhD
Department of Epidemiology and Public Health, Health Behaviour Research Centre, University College London, 1-19 Torrington Place, London, WC1E 7HB, UK

Ann McNeill, PhD
Department of Addictions, Institute of Psychiatry, Psychology and Neuroscience, King's College London, 4 Windsor Walk, Denmark Hill, London, SE5 8BB, UK

Word Count: 3,479
Abstract: 252

For peer review only

For peer review only - http://bmjopen.bmj.com/site/about/guidelines.xhtml

ABSTRACT

Objectives: To assess associations between smokers' strategies to minimise how much their smoking costs and cost of smoking among smokers across three social grades during a period of annual tax increases in England.
Design: Repeat cross-sectional.
Setting: England, May 2012-Dec 2016.
Participants: 16,967 adult smokers in 56 monthly surveys with nationally representative samples.
Measures and analysis: Generalised additive models (GAM) assessed associations between four cost-minimising strategies (changes in factory-made and roll-your-own (RYO) cigarette consumption, illicit and cross-border purchases) and cost of smoking (£/week), adjusting for inflation, age, gender, secular and seasonal trends.
Results Cost of smoking did not increase over time above inflation. Factory-made cigarette consumption decreased, while proportion of RYO and, to a much lesser extent, illicit and cross-border purchases increased. These trends were only evident in lowest social grade. Lowering factory-made cigarette consumption was associated with greater cost reductions (β=-12.65,95%CI=-12.84;-12.46) than lowering RYO consumption (β=-5.58,95%CI=-5.77;-5.38). Reductions in consumption accounted for 59% of variance in cost. Cross-border (β=-10.17,95%CI=-13.46;-6.88) and illicit tobacco purchases (β=-10.64,95%CI=-14.04;-7.25) were associated with lower costs, but due to low prevalence, accounted for only 0.2% of variation. Associations were similar across social grades, although weaker for illicit and cross-border purchases and stronger for consumption in higher social grades.
Conclusion During a period of annual tax increases, the cost of smoking above inflation did not change, while cost-minimising strategies increased, especially among more disadvantaged smokers. Reducing cigarette consumption and switching to RYO explained a large part of cost variation, while use of illicit and cross-border sources only a minor role.

Keywords: Smoking, costs, spending, expenditure, tobacco consumption, roll-your-own tobacco, illicit tobacco, cross-border purchasing

STRENGTHS AND LIMITATIONS OF THIS STUDY

Strengths
- This study used monthly data on a 4.5 year period from a large nationally representative sample of smokers across social grades.
- Data allowed comparison of the contribution of multiple cost minimising strategies to the cost of smoking.

Limitations
- Illicit and cross-border purchases were measured as any purchases over the past six months, and the variables may therefore not reflect the frequency of the use of these sources.
- The data did not contain information on usual brand of cigarettes, and we therefore did not take brand switching into account.
- We cannot rule out selective quitting, which may have affected the observed trend in cost of smoking if smokers who spend less are be more likely to quit than those who spend more.

For peer review only - http://bmjopen.bmj.com/site/about/guidelines.xhtml

INTRODUCTION

Increasing taxes on tobacco is considered among the most effective ways of reducing smoking prevalence.(1) In line with economic theory,(2) the resulting increased costs of smoking may provoke quit attempts, reduce smoking consumption, and deter uptake.(3, 4) Notably, tax increases are among the few interventions to show greater effectiveness in lower, compared to higher, socioeconomic strata,(5) which is important given the association between smoking and disadvantage in England(6) and other countries with a mature smoking epidemic.(7) The effectiveness of tax increases may however be diminished by the increased availability of cheap tobacco(8, 9) and cost-minimising strategies that smokers develop in response.(10, 11)

Estimates show that between half and three quarters of all smokers apply cost-minimising strategies(10-14) such as cutting down cigarette consumption, switching to a lower priced brand, switching to roll-your-own (RYO) tobacco, or evading or avoiding tobacco taxation by changing the source of purchase to illicit or cross-border/duty free sources, respectively (collectively known as non-UK duty-paid).(8, 10-13) US smokers who use one or more cost-minimising strategy have been shown to significantly reduce their costs, on average by 22%.(14, 15) Moreover, the use of cost-minimising strategies shows social patterning(16) with smokers of lower socioeconomic status (SES) being more likely to smoke RYO cigarettes and use cheaper factory-made cigarette brands,(12, 17) and smokers of higher SES being more likely to purchase untaxed tobacco products.(12, 18) The relative importance of different strategies however remains to be quantified, as well as the extent to which strategies differentially affect the cost of smoking among different socioeconomic groups.

The current study was set in the UK, a country that has among the highest tobacco tax rates worldwide.(19) In 2012-2016, taxes on all tobacco products increased above inflation nationwide in March of each year, by 5% in 2012, and 2% in 2013-2015. In 2016, a differential tax of 5% for RYO tobacco and 2% for factory-made cigarettes was applied.(20) Between 2002 and 2014, the proportion of UK smokers who used at least some RYO tobacco increased substantially, from 30 to 45%.(8) With RYO prices (per stick) being less than half of those of factory-made cigarettes,(8, 9) switching to RYO seems effective in lowering the cost of smoking. Between 2002 and 2014, use of tobacco from non-UK duty-paid sources remained consistent or even decreased,(8, 21) but increased slightly after 2015.(21) The extent to which the use of non-UK duty-paid tobacco contributes to mitigating the costs of smoking, and its relative importance compared with RYO tobacco, has not been previously established, especially across different social grade levels.

This study examined the extent to which the use of cost-minimising strategies allowed smokers from different social grades in England to minimise the actual cost of smoking in 2012-2016. We used data from the Smoking Toolkit Study (STS), which is a repeated cross-sectional monthly survey of the English population. The use of the STS allowed for the study of much more detailed trends and comprehensive measures of illicit tobacco use, in recent nationally representative data.
Specifically, the aims of this study were:

1. to describe trends in the cost of smoking (smokers' self-reported weekly spend on tobacco) and use of cost-minimising strategies between 2012 and 2016 overall and by social grade;
2. to assess in the general smoking population and across social grades, the association of cost of smoking with:
    a. the number of cigarettes smoked per week, both factory-made and RYO;
    b. and the purchase of non-UK duty-paid tobacco by means of purchase from either illicit or cross-border sources.

For peer review only - http://bmjopen.bmj.com/site/about/guidelines.xhtml

METHODS

Data and study population

Data were collected as part of the ongoing STS, a national repeated cross-sectional survey of tobacco use in the general population of England. Each month, a new sample of approximately 1700 adults aged ≥16 years is selected using a form of random location sampling. Individuals complete a face-to-face computer-assisted household interview survey with a trained interviewer. The STS samples have been shown to be nationally representative in their sociodemographic composition and proportion of smokers. Full details of the STS methods have been described elsewhere.(22) Ethical approval was granted by the University College London ethics committee.

We used data from 56 monthly waves from May 2012 to December 2016. May 2012 was selected as a starting point, as information on source of tobacco purchase was first measured from this wave. Out of a total of 97,074 respondents, we excluded non-smokers (N=78,184) and respondents with missing smoking status (N=68). Of 18,822 smokers, respondents with missing information on age (N=66) and respondents under 18 years of age (N=259) were excluded, because they could not legally purchase tobacco. We excluded respondents with implausible values for cost of smoking (N=837, assumptions for plausible spending are described below), smokers who did not report their weekly spend on tobacco (N=455), and smokers who did not report their cigarette consumption, or reported it to be zero (N=319). We included 16,967 current daily and non-daily cigarette smokers.

Measurements

The cost of smoking was measured as self-reported weekly spending on tobacco. Respondents were asked the following open-ended question: “On average about how much per week do you think you spend on cigarettes or tobacco?”. The cost of smoking was adjusted for inflation using Consumer Prices Index (CPI) data of all items from the Office for National Statistics (23), with December 2016 as the reference. Only smokers who adhered to three liberal assumptions of plausible levels of consumption and expenditure per week were included in the analysis, which led to the exclusion of 4.7% of smokers (N=837). The three assumptions included: 1) smokers smoke a maximum of 560 cigarettes per week (N=8), 2) spending does not exceed 280 pounds per week (N=7), and 3) single cigarettes cost between £0.05 and £1 (N=830).

Cost-minimising strategies included 1) reducing consumption of factory-made, 2) reducing consumption of RYO cigarettes (i.e. a cheap alternative for factory-made cigarettes), non-UK duty paid tobacco from 3) illicit sources, and 4) cross border sources. Factory-made and RYO cigarette consumption were treated as separate continuous variables in all analyses and were expressed in cigarettes per week. Respondents estimated for both factory-made and RYO cigarettes how many cigarettes they smoked per week. For exclusive factory-made cigarette users, RYO consumption levels were zero, and for exclusive RYO users, factory-made cigarette consumption levels were zero.

Purchase from illicit sources was measured as self-reported use of any of the following sources of tobacco at least once in the last 6 months: under the counter (from newsagent, off-license, or corner shop), pub (somebody comes around selling cheap), people who sell cheap cigarettes on the street, people in the local area who are a trusted source of cheap cigarettes, or cheap from friends. Cross-border purchasing was measured as self-reported use of cigarettes purchased abroad at least once in the last 6 months. Both were measured as dichotomous variables. Duty free sources within the UK were not specified as a response option and some respondents may have included these in their definition of cross-border sources.

For peer review only - http://bmjopen.bmj.com/site/about/guidelines.xhtml

Sociodemographic characteristics measured were gender, age and social grade. Social grade distinguished three categories: *low*: non-working class and (manual) working class (National Readership Survey (NRS) social grades D and E), *middle*: skilled working class and lower middle class (NRS grade C), and *high*: middle class and upper middle class (NRS grades A and B).

Time was measured in months throughout the study period. To control for seasonality (month-of-year effects), the month within the year ('calendar month') was coded as January=1 to December=12.

Statistical analysis

Data were analysed in R V.3.3.2. The analysis plan was registered on the Open Science Framework prior to data analysis (https://osf.io/ju6tf/). All data and analyses were unweighted and exact prevalence statistics will, therefore, differ marginally from previous STS publications (for more details, see Fidler et al.(22)). Descriptive statistics are given for the overall sample and stratified by social grade. Trends are graphically described and linear trends were tested using univariate generalised linear models.

Generalised additive models (GAMs) were used to assess the association between cost-minimising strategies and cost of smoking. GAMs are a type of generalised linear model that allow more sophisticated control for non-linear processes, in this case secular and seasonal trends, than standard linear regression models.(24) The natural log of smoking cost was used, in order to achieve a normal distribution. Results are presented as $100\cdot\beta$, which in this log-level model can be interpreted as the expected % change in cost of smoking for a unit increase in the covariate. All models included cyclic cubic regression splines for time (maximum of 5 knots; one for each year) and month within the year (maximum of 12 knots; one for each month). There was no evidence of autocorrelation between time periods according to the autocorrelation function (ACF) and partial ACF, and both the Durbin-Watson test and Breusch-Godfrey test were not statistically significant (p-values respectively 0.26 and 0.42).

Model 1 included socio-demographic characteristics (age, gender, and social grade). Models 2 to 5 included each cost-minimising strategy separately, adjusting for socio-demographic characteristics. The fully adjusted model (Model 6) was stratified according to social grade. We assessed for effect modification by social grade, by testing interaction between social grade and cost-minimising strategies. Interaction was also tested between cost-minimising strategies and time, in order to assess whether the influence of these strategies on cost changed over time.

We performed two sensitivity analyses using Model 6. First, factory-made cigarette and RYO cigarette consumption were replaced by total cigarette consumption and RYO proportion of that total. Second, the cost per cigarette was used as the outcome instead of cost of smoking per week.

RESULTS

Table 1 presents the description of the study population. Factory-made cigarette consumption was lower in the low social grade, while the consumption of RYO tobacco was higher. Illicit sources were more often used in the low social grade, while cross-border purchases were more common in the high social grade. For smokers in the middle social grade, figures for all four price minimising strategies and for reported cost of smoking were in between those of smokers in the low and high social grade.

For peer review only - http://bmjopen.bmj.com/site/about/guidelines.xhtml

Figure 1 shows the trends in cost of smoking, consumption, and use of illicit and cross-border sources of tobacco. No significant linear trend was found in the cost of smoking (increase of £0.18 per year, 95%CI-0.06 to 0.42, p=0.146). The number of RYO cigarettes consumed per week did not significantly change over time (-0.37 cigarettes per year, 95%CI -1.02 to 0.27, p=0.253), but there was a significant linear decreasing trend in factory-made cigarette consumption (-0.93 cigarettes per year, 95%CI -1.65 to -0.21, p=0.013). Therefore, the proportion of RYO within total cigarette consumption increased (+0.75% per year, 95%CI 0.13 to 1.38, p=0.020, data not shown in Figure 1). We found increasing trends in use of illicit sources (+0.49% per year, 95%CI 0.11 to 0.86, p=0.012) and cross-border purchase (+0.52% per year, 95%CI 0.17 to 0.88, p=0.005). A post-hoc analysis showed that cross-border purchasing mostly increased in the second half of 2016, but not significantly up to July 2016 (+0.15 per year, 95%CI -0.20 to 0.50, p=0.389). Trends by social grade are presented in Supplementary Figure 1. Linear tests showed that the trends observed in the total population were stronger, and only significant, among smokers in the low social grade.

Table 2 presents the associations between cost-minimising strategies and smoking cost. Cost of smoking was higher with increasing age, and higher in the low social grade than in the high social grade. Model 6 shows that most of the difference between the high and low social grades were attenuated by consumption or source of purchase. When controlling for consumption of factory-made cigarettes in Model 6, a decrease of ten RYO cigarettes per week was associated with 5.58% lower costs of smoking (95%CI:-5.38;-5.77). Controlling for RYO consumption, a decrease in consumption of ten factory-made cigarettes was associated with a 12.65% decrease in costs (95%CI:-12.46;-12.84). Both the use of illicit and cross-border sources of tobacco reduced the cost of smoking by around 10%. Due to their low prevalence, use of illicit and cross-border sources combined accounted for only 0.2% of variation in cost of smoking, while factory-made cigarette consumption and RYO consumption accounted for 50% and 9% of the variation in cost of smoking, respectively.

Table 3 shows the associations between cost-minimising strategies and the cost of smoking, by social grade. The same patterns were found in all three groups of social grade, with decreased consumption levels, and use of illicit and cross-border sources all associated with lower cost of smoking although associations between illicit and cross-border sources of tobacco and cost of smoking tended to be weaker and did not reach statistical significance in the high social grade. Associations of cigarette and RYO consumption with cost tended to be stronger among individuals in the high social grade compared with the low social grade, indicating that they smoke more expensive products.

In Table 4 we tested the interaction between time and cost-minimising strategies to assess whether the influence of these strategies on cost changed over time. The association between smoking cost and factory-made cigarette consumption grew stronger over time. Interactions with RYO consumption, use of illicit and cross-border sources of tobacco did not reach statistical significance.

Results for the sensitivity analyses are presented in the Supplementary Tables. Increasing the proportion of RYO cigarettes within total cigarette consumption was associated with a decrease in cost of smoking of 70.80% when 0% RYO was compared with 100% RYO (95%CI -72.80 to -68.94) (Supplementary Table 1). Note that these results were not corrected for the amount of tobacco that used for a RYO cigarette vs a manufactured cigarette. The price per cigarette (Supplementary Table 2) increased with a decrease in factory-made and RYO cigarette consumption. It decreased (around 12%) with the use of either illicit or cross-border sources.

DISCUSSION

For peer review only - http://bmjopen.bmj.com/site/about/guidelines.xhtml

Key findings

Reported cost of smoking in England did not increase over time above the rate of inflation, despite above-inflation tax increases. Factory-made cigarette consumption decreased, while the proportion of RYO and, to a much lesser extent, illicit and cross-border purchases increased. These trends were only evident in lowest social grade. Lowering factory-made cigarette consumption was associated with greater cost reductions than lowering RYO cigarette consumption. Consumption reduction accounted for 59% of variance. Cross-border and illicit tobacco purchases were associated with lower costs, but due to low prevalence, accounted for only 0.2% of variation in cost. Associations were similar across social grades, although illicit sources reduced the cost more strongly in smokers from low social grades than smokers from high social grades.

Limitations

The results of this study should be interpreted in light of the following limitations. Use of illicit and cross-border sources was measured dichotomously over a time frame of six months preceding the interview. This may have biased the results in two ways. First, we lacked information on the frequency of use of these sources. Because any one-time use is counted as using illicit/cross-border sources and the prevalence may therefore not represent, and likely overestimate, the proportion of not full duty-paid purchases out of total tobacco purchases. However, our prevalence rates are comparable with findings from the ITC UK data of 2010/2011 and 2014, in which the source of last purchase was measured.(8, 25) Second, the cost of smoking was measured over an average week, a much smaller timeframe than six months. Associations between smoking cost and tobacco sources may be diluted as a result of non-differential misclassification and the contribution to the cost of smoking may be larger than portrayed. However, given the very small share of 0.2% according to the current analysis, a substantially large share is unlikely.

The data did not contain information on usual brand. The associations found for the studied cost-minimising strategies may still be confounded by brand switching, as brand switching and the studied strategies are likely to co-occur.(10) Brand switching is a commonly used cost-minimising strategy.(10, 13) Choice of brand may have considerable impact on the cost of smoking, due to undershifting; the tobacco industry's strategy to divide tax increases disproportionately among different price segments.(9, 26) In general, undershifting caused low priced brands to have remained cheap, while prices of premium brands have increased.(9, 26, 27)

This study covers a period in which tobacco taxes increased above inflation annually, but the cost of smoking did not significantly increase above inflation rates. This may reflect a lack of effect of tax increases on the actual retail price of tobacco due to undershifting.(9, 26-28) The lack of an increasing trend may however also represent selective quitting. If smokers who spend more on tobacco are hit harder by a tax increase, they may be more likely to quit in response to increasing tax. The remaining smokers may therefore be those with lower levels of spending to begin with.

Interpretation

Our results are in line with previous findings that RYO cigarettes are much cheaper than factory-made cigarettes(9) as we found that switching from factory-made to RYO cigarettes is an effective cost-mitigating strategy. In our data, the proportion of RYO use increased over time. This increase has been observed since the early 2000's(28) and appears to continue over time.(8) Proportionally increased use is likely to be a response to an increasing gap in prices between factory-made and RYO.(9) Switching to RYO may have serious public health consequences, as smokers using RYO tobacco are much less sensitive to further price increases.(29) Moreover, smokers from lower social

For peer review only - http://bmjopen.bmj.com/site/about/guidelines.xhtml

grades are more likely to use RYO tobacco(28), which makes the increasing price gap likely to contribute to growing socioeconomic inequalities in smoking.(12) In order to encourage smoking cessation across social grades, taxes on RYO tobacco need to increase to the same level as factory-made cigarettes, which may be achieved through continued larger increases in RYO tobacco taxes.(8, 17, 30)

We found a small increase in the proportion of smokers who reported purchasing tobacco from illicit or cross-border sources. This is in line with an overview by Rowell et al., showing that the most reliable information on illicit tobacco does not show dramatic increases in use.(31) An analysis of 2002-2014 UK ITC data showed no increases in use of self-reported sources outside the UK or from informal sellers.(8) Illicit trade in cigarettes, measured by the tax gap between consumption and sales of tobacco, decreased from 16% in 2005-2006 to 8% in 2014-2015.(21) However, between 2015-16 and 2016-17, the tax gap increased to 15%,(21) which this paper reflects. The increase in tax gap in recent years seems mainly due to a decline in consumption rather than a growth in illicit trade.(21) The tax gap for RYO tobacco strongly declined from 60% in 2005 to 28% in 2017.(21)

We found that, in line with previous findings,(8, 12, 18) individuals from lower social grades were less likely to purchase tobacco abroad and more likely to use illicit sources, than smokers from higher social grades. Although statistical power was limited, associations between consumption levels and costs tended to be stronger among individuals in the high social grade compared with the low social grade. This may be because smokers in lower social grades are more likely to use lower priced brands,(28) for which the reduction of consumption has a smaller effect on the total cost of smoking. The association between illicit sources and the cost of smoking tended to be stronger in smokers from lower social grades, which may be explained by the frequency of using these sources, if smokers from lower social grades use illicit sources on a more regular basis.

Implications

In order to have actual costs of smoking increase above inflation, this study supports larger tax increases. However, tobacco taxation policies need to be designed in a way that takes industry strategies across brand segments and product types into account.(9, 26) As previously called for,(8, 9, 17, 26, 28, 30) this study makes a strong case for continued higher relative tax increases on RYO tobacco products compared with factory-made cigarettes. Other strategies may include maximising specific taxation, strong minimum price policies, plain packaging (already in effect in the UK), restricting brands to one variant and preventing the introduction of new brands.(9, 26, 32)

Although illicit tobacco formed only a minor threat to the costliness of smoking, a continued increase is undesirable. Action at the national and international level, including effective implementation of the FCTC protocol to eliminate illicit trade, has potential to reduce illicit trade in the near future.(33-35)

Conclusions

At a time when tax increases were designed to raise the cost of tobacco 2-5% above inflation annually, weekly spend on tobacco by smokers in England did not change above inflation. Our study showed that smokers commonly reduce consumption and switch to hand-rolled tobacco, particularly those of more disadvantaged social grades, but that the use of illicit and cross-border sources of tobacco was much less common and hardly contributed to total expenditure on tobacco. Strong future tobacco taxation policies are needed, that take industry strategies across brand segments and product types into account.

For peer review only - http://bmjopen.bmj.com/site/about/guidelines.xhtml

For peer review only

For peer review only - http://bmjopen.bmj.com/site/about/guidelines.xhtml

Funding

This work was supported by the National Institute for Health Research Public Health Research (project number 13/43/58). The Smoking Toolkit Study is currently primarily funded by Cancer Research UK (C1417/A22962; C44576/A19501) and has previously also been funded by Pfizer, GSK, and the Department of Health. Department of Health Disclaimer: The views and opinions expressed therein are those of the authors and do not necessarily reflect those of the Public Health Research programme, NIHR, NHS or the Department of Health.

No funders had any involvement in the design of the study, the analysis or interpretation of the data, the writing of the report, or the decision to submit the paper for publication.

Competing interests

RW undertakes consultancy and research for and receives travel funds and hospitality from manufacturers of smoking cessation medications. EB and JB have received unrestricted research funding from Pfizer. MK, TP, AG, and AM have no interests to declare.

Author contributions

JB, AM and RW conceived and designed the study. BC provided access to data on campaign expenditure. MAGK prepared and analysed the data, interpreted the findings and drafted the manuscript. AM, TP, JB, RW, EB and AG contributed to the interpretation of the data and provided critical revisions to the manuscript. All authors approved the final version of the paper for submission.

Data sharing statement

For access to the Smoking Toolkit Study please contact Dr Jamie Brown, jamie.brown@ucl.ac.uk.

For peer review only

For peer review only - http://bmjopen.bmj.com/site/about/guidelines.xhtml

REFERENCES

1. World Health Organization. WHO report on the gobal tobacco epidemic, 2008: The MPOWER package. Geneva; 2008 2008.
2. Phlips L. Applied Consumption Analysis: Advanced Textbooks in Economics: Elsevier; 2014.
3. Chaloupka FJ, Yurekli A, Fong GT. Tobacco taxes as a tobacco control strategy. Tobacco Control. 2012;21(2):172-80.
4. Biener L, Aseltine Jr RH, Cohen B, Anderka M. Reactions of adult and teenaged smokers to the Massachusetts tobacco tax. American Journal of Public Health. 1998;88(9):1389-91.
5. Brown T, Platt S, Amos A. Equity impact of population-level interventions and policies to reduce smoking in adults: a systematic review. Drug and alcohol dependence. 2014;138:7-16.
6. Hiscock R, Bauld L, Amos A, Platt S. Smoking and socioeconomic status in England: the rise of the never smoker and the disadvantaged smoker. Journal of Public Health. 2012;34(3):390-6.
7. Bosdriesz JR, Willemsen MC, Stronks K, Kunst AE. Socioeconomic inequalities in smoking cessation in 11 European countries from 1987 to 2012. J Epidemiol Community Health. 2015:jech-2014-205171.
8. Partos TR, Gilmore AB, Hitchman SC, Hiscock R, Branston JR, McNeill A. Availability and use of cheap tobacco in the UK 2002-2014: Findings from the International Tobacco Control Project. Nicotine & Tobacco Research. 2017:ntx108.
9. Hiscock R, Branston JR, McNeill A, Hitchman SC, Partos TR, Gilmore AB. Tobacco industry strategies undermine government tax policy: evidence from commercial data. Tobacco Control. 2017:tobaccocontrol-2017-053891.
10. Choi K, Hennrikus D, Forster J, St Claire AW. Use of price-minimizing strategies by smokers and their effects on subsequent smoking behaviors. Nicotine & Tobacco Research. 2012;14(7):864-70.
11. Licht AS, Hyland AJ, O'Connor RJ, Chaloupka FJ, Borland R, Fong GT, et al. How do price minimizing behaviors impact smoking cessation? Findings from the International Tobacco Control (ITC) Four Country Survey. International journal of environmental research and public health. 2011;8(5):1671-91.
12. Licht AS, Hyland AJ, O'Connor RJ, Chaloupka FJ, Borland R, Fong GT, et al. Socio-economic variation in price minimizing behaviors: findings from the International Tobacco Control (ITC) Four Country Survey. International journal of environmental research and public health. 2011;8(1):234-52.
13. White VM, Gilpin EA, White MM, Pierce JP. How do smokers control their cigarette expenditures? Nicotine & tobacco research. 2005;7(4):625-35.
14. Xu X, Pesko MF, Tynan MA, Gerzoff RB, Malarcher AM, Pechacek TF. Cigarette price-minimization strategies by US smokers. American journal of preventive medicine. 2013;44(5):472-6.
15. Pesko MF, Licht AS, Kruger JM. Cigarette price minimization strategies in the United States: price reductions and responsiveness to excise taxes. nicotine & tobacco research. 2013;15(11):1858-66.
16. Pesko MF, Kruger J, Hyland A. Cigarette price minimization strategies used by adults. American journal of public health. 2012;102(9):e19-e21.
17. Young D, Wilson N, Borland R, Edwards R, Weerasekera D. Prevalence, correlates of, and reasons for using roll-your-own tobacco in a high RYO use country: findings from the ITC New Zealand survey. Nicotine Tob Res. 2010;12(11):1089-98.
18. Nagelhout GE, van den Putte B, Allwright S, Mons U, McNeill A, Guignard R, et al. Socioeconomic and country variations in cross-border cigarette purchasing as tobacco tax avoidance strategy. Findings from the ITC Europe Surveys. Tobacco control. 2013:tobaccocontrol-2012-050838.
19. World Health Organization. WHO report on the global tobacco epidemic 2015: raising taxes on tobacco: World Health Organization; 2015.

For peer review only - http://bmjopen.bmj.com/site/about/guidelines.xhtml

20. Action on Smoking and Health. Timeline of changes in tobacco taxation in the UK from 1993 to the present 2017 [cited 2017. Available from: http://ash.org.uk/information-and-resources/taxation-illicit-trade/taxation/ash-analysis-of-tobacco-tax-increases-in-the-united-kingdom/.
21. HM Revenue & Customs. Measuring tax gaps 2017 edition. Tobacco tax gap estimates for 2016-17.; 2017.
22. Fidler JA, Shahab L, West O, Jarvis MJ, McEwen A, Stapleton JA, et al. 'The smoking toolkit study': a national study of smoking and smoking cessation in England. BMC Public Health. 2011;11(1):1.
23. CPI INDEX 00: ALL ITEMS 2015=100, (2017).
24. Wood SN. Generalized additive models: an introduction with R: Chapman and Hall/CRC; 2017.
25. Guindon GE, Driezen P, Chaloupka FJ, Fong GT. Cigarette tax avoidance and evasion: findings from the International Tobacco Control Policy Evaluation Project. Tobacco control. 2013:tobaccocontrol-2013-051074.
26. Gilmore AB, Tavakoly B, Taylor G, Reed H. Understanding tobacco industry pricing strategy and whether it undermines tobacco tax policy: the example of the UK cigarette market. Addiction. 2013;108(7):1317-26.
27. Marsh L, Cameron C, Quigg R, Hoek J, Doscher C, McGee R, et al. The impact of an increase in excise tax on the retail price of tobacco in New Zealand. Tobacco control. 2015:tobaccocontrol-2015-052259.
28. Gilmore AB, Tavakoly B, Hiscock R, Taylor G. Smoking patterns in Great Britain: the rise of cheap cigarette brands and roll your own (RYO) tobacco. Journal of public health. 2015;37(1):78-88.
29. Tait P, Rutherford P, Saunders C. Do consumers of manufactured cigarettes respond differently to price changes compared with their Roll-Your-Own counterparts? Evidence from New Zealand. Tobacco control. 2013:tobaccocontrol-2013-051010.
30. Young D, Yong H-H, Borland R, Shahab L, Hammond D, Cummings KM, et al. Trends in roll-your-own smoking: findings from the ITC Four-Country Survey (2002–2008). Journal of environmental and public health. 2012;2012.
31. Rowell A, Evans-Reeves K, Gilmore AB. Tobacco industry manipulation of data on and press coverage of the illicit tobacco trade in the UK. Tobacco control. 2014;23(e1):e35-e43.
32. Chaloupka FJ. Maximizing the public health impact of alcohol and tobacco taxes. American journal of preventive medicine. 2013;44(5):561-2.
33. McNeill A, Iringe-Koko B, Bains M, Bauld L, Siggens G, Russell A. Countering the demand for, and supply of, illicit tobacco: an assessment of the 'North of England Tackling Illicit Tobacco for Better Health'Programme. Tobacco control. 2013:tobaccocontrol-2013-050957.
34. HM Revenue & Customs. Customs (2015). Tackling illicit tobacco: From leaf to light 2017 [
35. World Health Organization. Protocol to eliminate illicit trade in tobacco products. 2013.

For peer review only

For peer review only - http://bmjopen.bmj.com/site/about/guidelines.xhtml

FIGURES AND TABLES

Table 1: Description of socio-demographics, cost-minimising strategies, and cost of smoking in the overall population of smokers and by social grade.

	Overall population	Social grade		
		**Low (N=7,032)**	**Middle (N=8,138)**	**High (N=1,797)**
Age distribution, % (95%CI)				
18 – 24	18.2 (17.6 to 18.8)	18.7 (17.8 to 19.6)	19.4 (18.6 to 20.3)	11.1 (9.8 to 12.7)
25 – 34	20.8 (20.2 to 21.4)	21.6 (20.6 to 22.5)	20.8 (19.9 to 21.6)	17.6 (15.9 to 19.4)
35 – 44	17.2 (16.7 to 17.8)	16.8 (16.0 to 17.7)	17.2 (16.4 to 18.0)	19.1 (17.4 to 21.0)
45 – 54	17.1 (16.5 to 17.6)	16.1 (15.3 to 17.0)	17.5 (16.6 to 18.3)	18.9 (17.2 to 20.8)
55 – 64	13.9 (13.4 to 14.5)	14.0 (13.2 to 14.8)	13.2 (12.5 to 14.0)	16.7 (15.0 to 18.5)
65+	12.8 (12.3 to 13.3)	12.8 (12.1 to 13.6)	11.9 (11.2 to 12.7)	16.5 (14.9 to 18.3)
Gender, % (95%CI)				
Male	47.5 (46.8 to 48.3)	51.8 (50.6 to 53.0)	44.8 (43.7 to 45.8)	43.2 (40.9 to 45.5)
Female	52.5 (51.7 to 53.2)	48.2 (47.0 to 49.4)	55.2 (54.2 to 56.3)	56.8 (54.5 to 59.1)
Social grade, % (95%CI)				
Low	41.4 (40.7 to 42.2)	-	-	-
Middle	48.0 (47.2 to 48.7)	-	-	-
High	10.6 (10.1 to 11.1)	-	-	-
Factory-made cigarette consumption in cigarettes/week, mean (95%CI)	45.9 (45.1 to 46.7)	43.3 (42.0 to 44.6)	47.1 (45.8 to 48.3)	50.8 (48.3 to 53.4)
RYO cigarette consumption in cigarettes/week, mean (95%CI)	36.2 (35.4 to 37.0)	44.8 (43.4 to 46.2)	31.8 (30.8 to 32.9)	22.0 (20.1 to 24.0)
Use of illicit sources, % (95%CI)	8.2 (7.8 to 8.6)	9.8 (9.1 to 10.5)	7.5 (7.0 to 8.1)	5.0 (4.0 to 6.0)
Cross-border purchase, % (95%CI)	7.4 (7.0 to 7.8)	4.2 (3.8 to 4.7)	8.9 (8.3 to 9.6)	13.2 (11.7 to 14.8)
Cost of smoking in £/week, mean (95%CI)	23.3 (23.0 to 23.5)	22.8 (22.3 to 23.2)	23.5 (23.1 to 23.9)	24.1 (23.2 to 25.0)

Figure 1: Trends in cost of smoking, cigarette consumption, use of illicit sources, and cross-border purchase among smokers in England.

For peer review only

For peer review only - http://bmjopen.bmj.com/site/about/guidelines.xhtml

Table 2: Percentage difference in cost of smoking for socio-demographics and cost-minimising strategies from Generalised Additive Models (GAM).

	Percentage difference in cost of smoking (100β with 95%CI)		
	**Model 1** Baseline model	**Models 2 to 5** Adjusted for socio-demographics	**Model 6** All variables
Age			
Per 10 years increase	6.68 (5.94 to 7.43)		0.13 (-0.40 to 0.66)
Gender			
Male	ref		ref
Female	0.78 (-1.75 to 3.30)		0.11 (-1.67 to 1.90)
Social grade			
Low	ref		ref
Middle	-0.99 (-3.66 to 1.68)		1.44 (-0.45 to 3.34)
High	-6.93 (-11.25 to -2.59)		-0.91 (-4.00 to 2.17)
Factory-made cigarette consumption[a]			
Per 10 cigarettes decrease		-9.95 (-10.12 to -9.77)	-12.65 (-12.84 to -12.46)
RYO cigarette consumption[b]			
Per 10 cigarettes decrease		1.15 (0.91 to 1.39)	-5.58 (-5.77 to -5.38)
Use of illicit sources[c]			
No use of illicit sources in last 6 months		ref	ref
Used illicit sources in last 6 months		-7.23 (-11.84 to -2.62)	-10.17 (-13.46 to -6.88)
Cross-border purchase[d]			
No cross-border purchase in last 6 months		ref	ref
Cross-border purchase in last 6 months		-9.48 (-14.31 to -4.65)	-10.64 (-14.04 to -7.25)

RYO = roll-your-own
[a] 49.7% of variance in spending accounted for by factory-made cigarette consumption.
[b] 8.8% of variance in spending accounted for by RYO cigarette consumption.
[c] 0.1% of variance in spending accounted for by use of illicit sources of tobacco.
[d] 0.1% of variance in spending accounted for by use of cross-border sources of tobacco.

For peer review only

Table 3: Percentage difference in cost of smoking for socio-demographics and cost-minimising strategies from Generalised Additive Models (GAM), stratified by social grade.

	Social grade				
	Low (N=7,032)	Middle (N=8,138)		High (N=1,797)	
	100β with 95%CI	100β with 95%CI	P-value for interaction, middle vs low	100β with 95%CI	P-value for interaction, high vs low
Age					
Per 10 years increase	-0.18 (-0.95 to 0.59)	-0.14 (-0.65 to 0.92)		0.35 (-1.46 to 2.16)	
Gender					
Male	ref	ref		ref	
Female	0.65 (1.99 to 3.29)	-0.37 (-2.98 to 2.24)		-1.09 (-7.00 to 4.82)	
Factory-made cigarette consumption					
Per 10 cigarettes decrease	-11.36 (-11.64 to -11.08)	-13.19 (-13.47 to -12.92)	<0.001	-15.19 (-15.81 to -14.58)	<0.001
RYO cigarette consumption					
Per 10 cigarettes decrease	-4.73 (-4.99 to -4.47)	-6.07 (-6.37 to -5.76)	0.010	-7.74 (-8.54 to -6.95)	0.011
Use of illicit sources					
No use of illicit sources in last 6 months	ref	ref		ref	
Used illicit sources in last 6 months	-12.57 (-17.07 to -8.07)	-7.47 (-12.45 to -2.49)	0.027	-2.94 (-16.51 to 10.64)	0.059
Cross-border purchase					
No cross-border purchase in last 6 months	ref	ref		ref	
Cross-border purchase in last 6 months	-14.71 (-21.24 to -8.18)	-10.79 (-15.33 to -6.25)	0.194	-7.54 (-16.17 to 1.10)	0.035

RYO = roll-your-own

Note: All models were adjusted for all variables in the table.

Table 4: Increase per year in the % difference in cost of smoking for cost-minimising strategies from Generalised Additive Models (GAM)

	Increase in the % change in cost of smoking for each consecutive year (95%CI)	P-value for interaction
Factory-made cigarette consumption · time	-0.15 (-0.26 to 0.03)	0.012
RYO cigarette consumption · time	0.02 (-0.10 to 0.14)	0.740
Illicit sources · time	1.02 (-1.36 to 3.40)	0.402
Cross-border purchase · time	-0.14 (-2.55 to 2.26)	0.909

RYO = roll-your-own tobacco

Note: Models were adjusted for age, gender, social grade, and other cost-minimising strategies in the table.

For peer review only - http://bmjopen.bmj.com/site/about/guidelines.xhtml

For peer review only

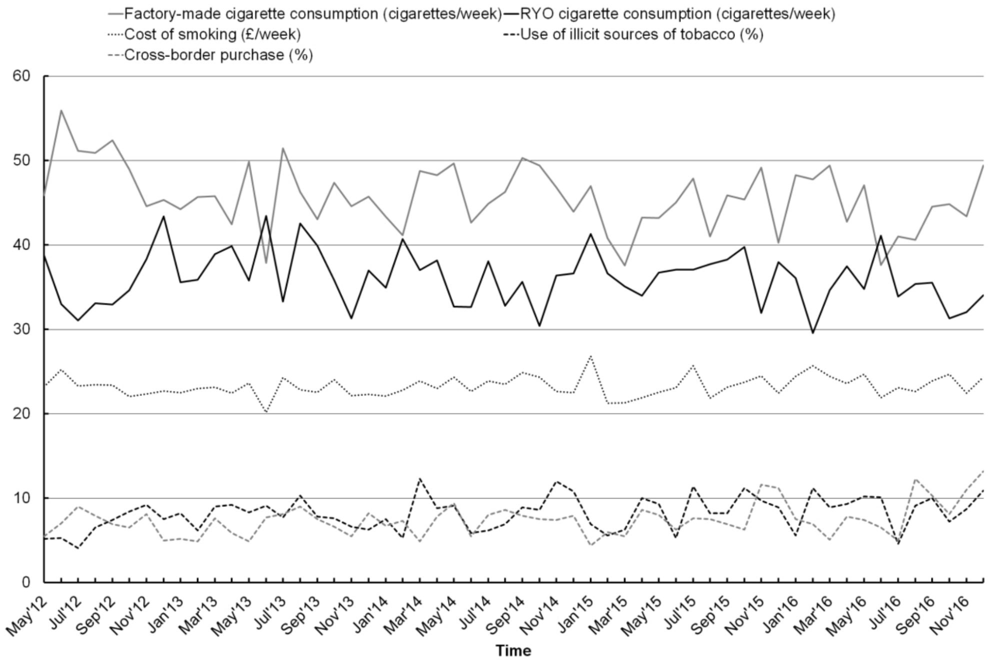

Trends in cost of smoking, cigarette consumption, use of illicit sources, and cross-border purchase among smokers in England.

198x132mm (300 x 300 DPI)

SUPPLEMENTARY FILES

Supplementary Table 1: Results from Generalised Additive Models (GAM) for cost of smoking, with total cigarette consumption and proportion of RYO use.

	**Percentage difference in cost of smoking (100β with 95%CI)**
	**Model 6**<br>Adjusted for other cost-minimising strategies
Age (per 10 years increase)	-0.27 (-0.79 to 0.26)
Gender	
Male	Ref
Female	1.22 (-0.56 to 2.99)
Social grade	
Low	ref
Middle	2.48 (0.60 to 4.37)
High	-0.76 (-3.83 to 2.30)
Total cigarette consumption (per 10 cigarettes decrease)	-9.62 (-9.78 to -9.45)
Proportion of RYO cigarette consumption[a]	-70.80 (-72.80 to -68.94)
Use of illicit sources	-10.48 (-13.75 to -7.21)
Use of cross-border sources	-9.65 (-13.03 to -6.27)

[a] Represents the difference between 0% RYO and 100% RYO cigarettes of total consumption.

Supplementary Table 2: Results from Generalised Additive Models (GAM) for price per cigarette.

	**Percentage difference in price per cigarette (100β with 95%CI)**
	**Model 6**<br>Adjusted for other cost-minimising strategies
Age (per 10 years increase)	-0.80 (-1.25 to -0.34)
Gender	
Male	Ref
Female	1.59 (0.06 to 3.11)
Social grade	
Low	ref
Middle	5.73 (4.11 to 7.35)
High	10.42 (7.78 to 13.06)
Factory-made cigarette consumption (per 10 cigarettes decrease)	0.77 (0.61 to 0.93)
RYO cigarette consumption (per 10 cigarettes decrease)	7.62 (7.45 to 7.78)
Use of illicit sources	-12.29 (-15.11 to -9.48)
Use of cross-border sources	-12.19 (-15.10 to -9.28)

For peer review only

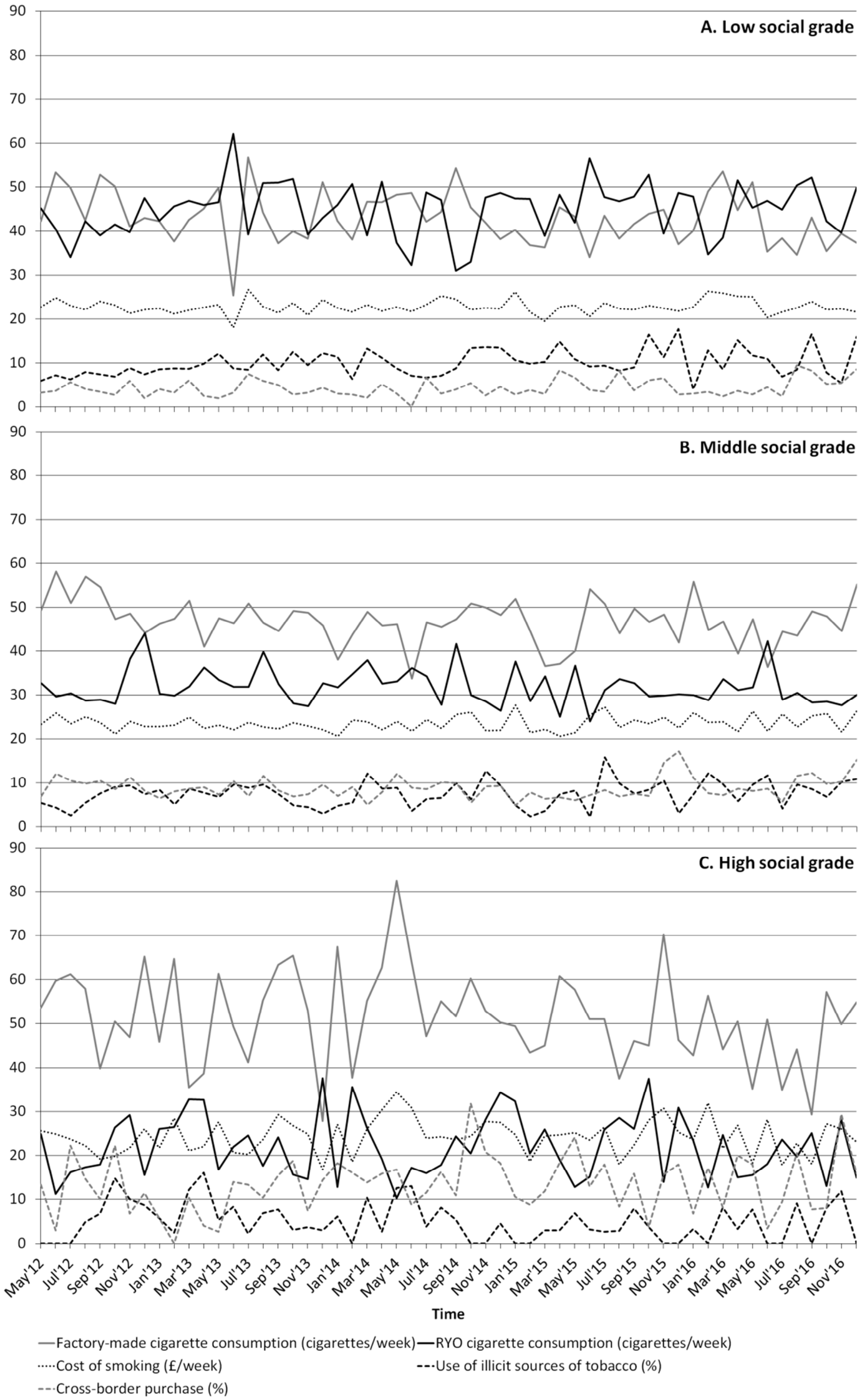

Supplementary Figure 1: Trends in cost of smoking, cigarette consumption, use of illicit sources, and cross-border purchase among smokers in England, stratified by social grade.

STROBE Statement—Checklist of items that should be included in reports of ***cross-sectional studies***

	Page no.	Item No	Recommendation
**Title and abstract**	2	1	(*a*) Indicate the study's design with a commonly used term in the title or the abstract
	2		(*b*) Provide in the abstract an informative and balanced summary of what was done and what was found
	**Introduction**		
Background/rationale	3	2	Explain the scientific background and rationale for the investigation being reported
Objectives	3	3	State specific objectives, including any prespecified hypotheses
	**Methods**		
Study design	4	4	Present key elements of study design early in the paper
Setting	4	5	Describe the setting, locations, and relevant dates, including periods of recruitment, exposure, follow-up, and data collection
Participants	4	6	(*a*) Give the eligibility criteria, and the sources and methods of selection of participants
Variables	4,5	7	Clearly define all outcomes, exposures, predictors, potential confounders, and effect modifiers. Give diagnostic criteria, if applicable
Data sources/ measurement	4	8*	For each variable of interest, give sources of data and details of methods of assessment (measurement). Describe comparability of assessment methods if there is more than one group
Bias	5	9	Describe any efforts to address potential sources of bias
Study size	4	10	Explain how the study size was arrived at
Quantitative variables	4,5	11	Explain how quantitative variables were handled in the analyses. If applicable, describe which groupings were chosen and why
Statistical methods	5	12	(*a*) Describe all statistical methods, including those used to control for confounding
	5		(*b*) Describe any methods used to examine subgroups and interactions
	4		(*c*) Explain how missing data were addressed
	N/A		(*d*) If applicable, describe analytical methods taking account of sampling strategy
	5		(*e*) Describe any sensitivity analyses
	**Results**		
Participants	N/A	13*	(a) Report numbers of individuals at each stage of study—eg numbers potentially eligible, examined for eligibility, confirmed eligible, included in the study, completing follow-up, and analysed
	N/A		(b) Give reasons for non-participation at each stage
	N/A		(c) Consider use of a flow diagram
Descriptive data	5,12	14*	(a) Give characteristics of study participants (eg demographic, clinical, social) and information on exposures and potential confounders
	4		(b) Indicate number of participants with missing data for each variable of interest
Outcome data	fig1	15*	Report numbers of outcome events or summary measures
Main results	6,13	16	(*a*) Give unadjusted estimates and, if applicable, confounder-adjusted

For peer review only - http://bmjopen.bmj.com/site/about/guidelines.xhtml
For peer review only

			estimates and their precision (eg, 95% confidence interval). Make clear which confounders were adjusted for and why they were included
	N/A		(*b*) Report category boundaries when continuous variables were categorized
	N/A		(*c*) If relevant, consider translating estimates of relative risk into absolute risk for a meaningful time period
Other analyses	6, Suppl. tables	17	Report other analyses done—eg analyses of subgroups and interactions, and sensitivity analyses
	**Discussion**		
Key results	6,7	18	Summarise key results with reference to study objectives
Limitations	7	19	Discuss limitations of the study, taking into account sources of potential bias or imprecision. Discuss both direction and magnitude of any potential bias
Interpretation	7,8	20	Give a cautious overall interpretation of results considering objectives, limitations, multiplicity of analyses, results from similar studies, and other relevant evidence
Generalisability	8	21	Discuss the generalisability (external validity) of the study results
	**Other information**		
Funding	9	22	Give the source of funding and the role of the funders for the present study and, if applicable, for the original study on which the present article is based

*Give information separately for exposed and unexposed groups.

Note: An Explanation and Elaboration article discusses each checklist item and gives methodological background and published examples of transparent reporting. The STROBE checklist is best used in conjunction with this article (freely available on the Web sites of PLoS Medicine at http://www.plosmedicine.org/, Annals of Internal Medicine at http://www.annals.org/, and Epidemiology at http://www.epidem.com/). Information on the STROBE Initiative is available at www.strobe-statement.org.

For peer review only

BMJ Open

Smokers' strategies across social grades to minimise the cost of smoking in a period with annual tax increases: Evidence from a national survey in England

Journal:	*BMJ Open*
Manuscript ID	bmjopen-2018-026320.R1
Article Type:	Research
Date Submitted by the Author:	18-Jan-2019
Complete List of Authors:	Kuipers, Mirte; Academic Medical Center, University of Amsterdam,<br>Partos, Timea; Kings College London, Addictions<br>Brown, Jamie; University College London, Psychology & Language Sciences<br>Beard, Emma; UCL,<br>Gilmore, Anna; Tobacco Control Research Group, UK Centre for Tobacco and Alcohol Studies, Department for Health, University of Bath<br>West, Robert; University College London<br>McNeill, Ann; King's College London, UK Centre for Tobacco Control Studies, National Addiction Centre, Institute of Psychiatry
**Primary Subject Heading**:	Smoking and tobacco
Secondary Subject Heading:	Epidemiology, Public health
Keywords:	EPIDEMIOLOGY, PREVENTIVE MEDICINE, PUBLIC HEALTH

SCHOLARONE™
Manuscripts

Smokers' strategies across social grades to minimise the cost of smoking in a period with annual tax increases: Evidence from a national survey in England

Mirte AG Kuipers, PhD, corresponding author
Department of Epidemiology and Public Health, Health Behaviour Research Centre, University College London, London, UK
Department of Public Health, Amsterdam Public Health Research Institute, Academic Medical Center – University of Amsterdam, P.O. Box 22660, 1100 DD, Amsterdam, The Netherlands
E-mail address: m.a.kuipers@amc.uva.nl

Timea R Partos, PhD
Department of Addictions, Institute of Psychiatry, Psychology and Neuroscience, King's College London, 4 Windsor Walk, Denmark Hill, London, SE5 8BB, UK

Jamie Brown, PhD
Research Department of Clinical, Educational and Health Psychology, University College London, 1-19 Torrington Place, London, WC1E 7HB, UK

Emma Beard, PhD
Research Department of Clinical, Educational and Health Psychology, University College London, 1-19 Torrington Place, London, WC1E 7HB, UK

Anna Gilmore, PhD
Tobacco Control Research Group, Department for Health, University of Bath, Claverton Down, Bath, BA2 7AY, UK

Robert West, PhD
Department of Epidemiology and Public Health, Health Behaviour Research Centre, University College London, 1-19 Torrington Place, London, WC1E 7HB, UK

Ann McNeill, PhD
Department of Addictions, Institute of Psychiatry, Psychology and Neuroscience, King's College London, 4 Windsor Walk, Denmark Hill, London, SE5 8BB, UK

For peer review only

Word Count: 3,937
Abstract: 274

ABSTRACT

Objectives: To assess associations between smokers' strategies to minimise how much their smoking costs and cost of smoking among smokers across three social grades during a period of annual tax increases in England.
Design: Repeat cross-sectional.
Setting: England, May 2012-Dec 2016.
Participants: 16,967 adult smokers in 56 monthly surveys with nationally representative samples.
Measures and analysis: Generalised additive models (GAM) assessed associations between four cost-minimising strategies (factory-made and roll-your-own (RYO) cigarette consumption levels, illicit and cross-border purchases) and cost of smoking (£/week). We adjusted for inflation rate, age, gender, and secular and seasonal trends.
Results Cost of smoking did not increase above the rate of inflation. Factory-made cigarette consumption decreased, while proportion of RYO and, to a much lesser extent, illicit and cross-border purchases increased. These trends were only evident in lowest social grade. Cost of smoking was 12.65% lower with consumption of 10 fewer factory-made cigarettes (95%CI=-12.84;-12.46), and 5.58% lower with consumption of 10 fewer RYO cigarettes (95%CI=-5.77;-5.38). Consumption levels accounted for 59% of variance in cost. Cross-border and illicit tobacco purchases were associated with 10.17% (95%CI=-13.46;-6.88) and 10.64% (95%CI=-14.04;-7.25) lower costs, respectively, but due to low prevalence, accounted for only 0.2% of variation. Associations were similar across social grades, although weaker for illicit and cross-border purchases and stronger for consumption in higher social grades compared with lower social grades.
Conclusion During a period of annual tax increases, the weekly cost of smoking did not increase above inflation. Cost-minimising strategies increased, especially among more disadvantaged smokers. Reducing cigarette consumption and switching to roll-your-own tobacco explained a large part of cost variation, while use of illicit and cross-border purchasing played only a minor role.

Keywords: Smoking, costs, spending, expenditure, tobacco consumption, roll-your-own tobacco, illicit tobacco, cross-border purchasing

STRENGTHS AND LIMITATIONS OF THIS STUDY

Strengths

- This study used monthly data on a 4.5 year period from a large nationally representative sample of smokers across social grades.
- Data allowed comparison of the contribution of multiple cost minimising strategies to the cost of smoking.

Limitations

- Illicit and cross-border purchases were measured as any purchases over the past six months, and the variables may therefore not reflect the frequency of the use of these sources.
- The data did not contain information on usual brand of cigarettes, and we therefore did not take brand switching into account.
- We cannot rule out selective quitting, which may have affected the observed trend in cost of smoking if smokers who spend less are be more likely to quit than those who spend more.

For peer review only - http://bmjopen.bmj.com/site/about/guidelines.xhtml

INTRODUCTION

Increasing taxes on tobacco is considered among the most effective ways of reducing smoking prevalence.(1) In line with economic theory,(2) the resulting increased costs of smoking may provoke quit attempts, reduce smoking consumption, and deter uptake.(3, 4) Notably, tax increases are among the few interventions to show greater effectiveness in lower, compared to higher, socioeconomic strata,(5) which is important given the association between smoking and disadvantage in England(6) and other countries with a mature smoking epidemic.(7) The effectiveness of tax increases may however be diminished by the increased availability of cheap tobacco(8, 9) and cost-minimising strategies that smokers develop in response.(10, 11)

Studies across high-income countries estimate that between half and three quarters of all smokers apply cost-minimising strategies(10-14) such as cutting down cigarette consumption, switching to a lower priced brand, switching to roll-your-own (RYO) tobacco, or evading or avoiding tobacco taxation by changing the source of purchase to illicit or cross-border/duty free sources, respectively (collectively known as non-UK duty-paid).(8, 10-13) US smokers who use one or more cost-minimising strategy have been shown to significantly reduce their costs, on average by 22%.(14, 15) Moreover, the use of cost-minimising strategies shows social patterning(16) with smokers of lower socioeconomic status (SES) being more likely to smoke RYO cigarettes and use cheaper factory-made cigarette brands,(12, 17) and smokers of higher SES being more likely to purchase untaxed tobacco products.(12, 18) The relative importance of different strategies however remains to be quantified, as well as the extent to which strategies differentially affect the cost of smoking among different socioeconomic groups.

The current study was set in the UK, where there has been a marked decrease in smoking prevalence (from 19.3% in 2012 to 15.5% in 2016(19)) and where some use of cost-minimising strategies has been demonstrated using data from the International Tobacco Control policy evaluation project.(8, 11, 12, 18) The UK has among the highest tobacco tax rates worldwide.(20) In 2012-2016, taxes on all tobacco products increased nationwide in March of each year, by 5% above inflation in 2012, and 2% above inflation in 2013-2015. In 2016, a differential tax of 5% above inflation for RYO tobacco and 2% above inflation for factory-made cigarettes was applied.(21) Between 2002 and 2014, the proportion of UK smokers who used at least some RYO tobacco increased substantially, from 30 to 45%.(8) With RYO prices (per stick) being less than half of those of factory-made cigarettes,(8, 9) switching to RYO seems effective in lowering the cost of smoking. Between 2002 and 2014, use of tobacco from non-UK duty-paid sources remained consistent or even decreased,(8, 22) but increased slightly after 2015.(22) The extent to which the use of non-UK duty-paid tobacco contributes to mitigating the costs of smoking, and its relative importance compared with RYO tobacco, has not been previously established, especially across different social grade levels.

This study examined the extent to which the use of cost-minimising strategies allowed smokers from different social grades in England to minimise the actual cost of smoking in 2012-2016. We used data from the Smoking Toolkit Study (STS), which is a repeated cross-sectional monthly survey of the English population. The use of the STS allowed for the study of much more detailed trends and comprehensive measures of illicit tobacco use, in recent nationally representative data.
Specifically, the aims of this study were:

1. to describe trends in the cost of smoking (smokers' self-reported weekly spend on tobacco) and use of cost-minimising strategies between 2012 and 2016 overall and by social grade;
2. to assess in the general smoking population and across social grades, the association of cost of smoking with:
    a. the number of cigarettes smoked per week, both factory-made and RYO;

For peer review only - http://bmjopen.bmj.com/site/about/guidelines.xhtml

b. and the purchase of non-UK duty-paid tobacco by means of purchase from either illicit or cross-border sources.

METHODS

Data and study population

Data were collected as part of the ongoing STS, a national repeated cross-sectional survey of tobacco use in the general population of England. Each month, a new sample of approximately 1700 adults aged ≥16 years is selected using a form of random location sampling. Individuals complete a face-to-face computer-assisted household interview survey with a trained interviewer. The STS samples have been shown to be nationally representative in their sociodemographic composition and proportion of smokers. Full details of the STS methods have been described elsewhere.(23) Ethical approval was granted by the University College London ethics committee.

We used data from 56 monthly waves from May 2012 to December 2016. May 2012 was selected as a starting point, as information on source of tobacco purchase was first measured from this wave. Out of a total of 97,074 respondents, we excluded non-smokers (N=78,184) and respondents with missing smoking status (N=68). Of 18,822 smokers, respondents with missing information on age (N=66) and respondents under 18 years of age (N=259) were excluded, because they could not legally purchase tobacco. We excluded respondents with implausible values for cost of smoking (N=837, assumptions for plausible spending are described below), smokers who did not report their weekly spend on tobacco (N=455), and smokers who did not report their cigarette consumption, or reported it to be zero (N=319). We included 16,967 current daily and non-daily cigarette smokers.

Patient and public involvement

This study involved secondary data analysis of existing data from the Smoking Toolkit Study (STS) surveys. Participants and public were not involved in the current study.

Measurements

The cost of smoking was measured as self-reported weekly spending (in £) on tobacco. Respondents were asked the following open-ended question: “On average about how much per week do you think you spend on cigarettes or tobacco?”. The cost of smoking was adjusted for inflation using Consumer Prices Index (CPI) data of all items from the Office for National Statistics (24), with December 2016 as the reference. Only smokers who adhered to three liberal assumptions of plausible levels of consumption and expenditure per week were included in the analysis, which led to the exclusion of 4.7% of smokers (N=837). The three assumptions included: 1) smokers smoke a maximum of 560 cigarettes per week (N=8), 2) spending does not exceed 280 pounds per week (N=7), and 3) single cigarettes cost between £0.05 and £1 (N=830).

Cost-minimising strategies included 1) reducing consumption of factory-made, 2) reducing consumption of RYO cigarettes (i.e. a cheap alternative for factory-made cigarettes(9)), non-UK duty paid tobacco from 3) illicit sources, and 4) cross border sources. Factory-made and RYO cigarette consumption were treated as separate continuous variables in all analyses and were expressed in cigarettes per week. Respondents estimated for both factory-made and RYO cigarettes how many cigarettes they smoked per week. For exclusive factory-made cigarette users, RYO consumption levels were zero, and for exclusive RYO users, factory-made cigarette consumption levels were zero.

For peer review only - http://bmjopen.bmj.com/site/about/guidelines.xhtml

Purchase from illicit sources was measured as self-reported use of any of the following sources of tobacco at least once in the last 6 months: under the counter (from newsagent, off-license, or corner shop), pub (somebody comes around selling cheap), people who sell cheap cigarettes on the street, people in the local area who are a trusted source of cheap cigarettes, or cheap from friends. Cross-border purchasing was measured as self-reported use of cigarettes purchased abroad at least once in the last 6 months. Both were measured as dichotomous variables. Duty free sources within the UK were not specified as a response option and some respondents may have included these in their definition of cross-border sources.

Sociodemographic characteristics measured were gender, age and social grade. Social grade was assigned by the interviewer based on the occupation of the chief income earner of the household and used the National Readership Survey classification system to distinguish three categories: *low*: non-working class and (manual) working class (National Readership Survey (NRS) social grades D and E), *middle*: skilled working class and lower middle class (NRS grade C), and *high*: middle class and upper middle class (NRS grades A and B).

Time was measured in months throughout the study period. To control for seasonality (month-of-year effects), the month within the year ('calendar month') was coded as January=1 to December=12.

Statistical analysis

Data were analysed in R V.3.3.2. The analysis plan was registered on the Open Science Framework prior to data analysis (https://osf.io/ju6tf/). All data and analyses were unweighted and exact prevalence statistics will, therefore, differ marginally from previous STS publications (for more details, see Fidler et al.(23)). Weighted analyses were performed as a post-hoc sensitivity analysis, and resulted in similar findings that would lead to the same conclusions.

Descriptive statistics are given for the overall sample and stratified by social grade. Trends are graphically described and linear trends were tested using univariate generalised linear models.

Generalised additive models (GAMs) were used to assess the association between cost-minimising strategies and cost of smoking. GAMs are a type of generalised linear model that allow more sophisticated control for non-linear processes, in this case secular and seasonal trends, than standard linear regression models.(25) The natural log of smoking cost was used, in order to achieve a normal distribution. Results are presented as $100 \cdot \beta$, which in this log-level model can be interpreted as the expected % difference in cost of smoking for a unit increase in the covariate. All models included cyclic cubic regression splines for time (maximum of 5 knots; one for each year) and month within the year (maximum of 12 knots; one for each month). There was no evidence of autocorrelation between time periods according to the autocorrelation function (ACF) and partial ACF, and both the Durbin-Watson test and Breusch-Godfrey test were not statistically significant (p-values respectively 0.26 and 0.42).

Model 1 included socio-demographic characteristics (age, gender, and social grade). Models 2 to 5 included each cost-minimising strategy separately, adjusting for socio-demographic characteristics. The fully adjusted model (Model 6) included all cost-minimising strategies plus socio-demographics. Model 6 was stratified according to social grade. We assessed effect modification by social grade, by testing interaction between social grade and cost-minimising strategies. Interaction was also tested between cost-minimising strategies and time, in order to assess whether the influence of these strategies on cost changed over time.

We performed two sensitivity analyses using Model 6. First, factory-made cigarette and RYO cigarette consumption were replaced by total cigarette consumption and RYO proportion of that total, as an

For peer review only - http://bmjopen.bmj.com/site/about/guidelines.xhtml

alternative way of measuring the use of RYO as a substitute for factory-made cigarettes and reflecting its relative cost. Second, the cost per cigarette was used as the outcome instead of cost of smoking per week, as an alternative way of measuring the cost of smoking, that is less dependent on the level of consumption. A post-hoc analysis of Model 6 was carried out in the dataset in which we did not exclude values of cost of smoking based on single cigarettes cost (N=17,789).

RESULTS

Table 1 presents the description of the study population. Factory-made cigarette consumption was lower in the low social grade, while the consumption of RYO tobacco was higher. Illicit sources were more often used in the low social grade, while cross-border purchases were more common in the high social grade. For smokers in the middle social grade, figures for all four price minimising strategies and for reported cost of smoking were in between those of smokers in the low and high social grade.

Figure 1 shows the trends in cost of smoking, consumption, and use of illicit and cross-border sources of tobacco. No significant linear trend was found in the cost of smoking (increase of £0.18 per year, 95%CI-0.06 to 0.42, p=0.146). The number of RYO cigarettes consumed per week did not significantly change over time (-0.37 cigarettes per year, 95%CI -1.02 to 0.27, p=0.253), but there was a significant linear decreasing trend in factory-made cigarette consumption (-0.93 cigarettes per year, 95%CI -1.65 to -0.21, p=0.013). This means that within total cigarette consumption (i.e., the sum of RYO and factory-made cigarettes), the proportion of RYO consumption increased (+0.75% per year, 95%CI 0.13 to 1.38, p=0.020, data not shown in Figure 1). We found increasing trends in use of illicit sources (+0.49% per year, 95%CI 0.11 to 0.86, p=0.012) and cross-border purchase (+0.52% per year, 95%CI 0.17 to 0.88, p=0.005). A post-hoc analysis showed that cross-border purchasing mostly increased in the second half of 2016, but not significantly up to July 2016 (+0.15 per year, 95%CI -0.20 to 0.50, p=0.389). Trends by social grade are presented in Supplementary Figure 1. Linear tests showed that the trends observed in the total population were stronger, and only significant among smokers in the low social grade.

Table 2 presents the associations between cost-minimising strategies and smoking cost. Cost of smoking was higher with increasing age, and higher in the low social grade than in the high social grade. In the fully adjusted model (Model 6), the spline term for trend over the years was significant (p=0.007), while the trend within years was not (p=0.233; not presented in table). Model 6 shows that most of the difference between the high and low social grades were attenuated by consumption or source of purchase. When controlling for consumption of factory-made cigarettes in Model 6, a decrease of ten RYO cigarettes per week was associated with 5.58% lower costs of smoking (95%CI:-5.38;-5.77). Controlling for RYO consumption, a decrease in consumption of ten factory-made cigarettes was associated with a 12.65% decrease in costs (95%CI:-12.46;-12.84). Both the use of illicit and cross-border sources of tobacco reduced the cost of smoking (Illicit: -10.17%, 95%CI: -13.46 to -6.88; cross-border: -10.64%, 95%CI: -14.04 to -7.25). Due to their low prevalence, use of illicit and cross-border sources combined accounted for only 0.2% of variation in cost of smoking, while factory-made cigarette consumption and RYO consumption accounted for 50% and 9% of the variation in cost of smoking, respectively.

Table 3 shows the associations between cost-minimising strategies and the cost of smoking, by social grade. The same patterns were found in all three groups of social grade, with decreased consumption levels, and use of illicit and cross-border sources all associated with lower cost of smoking although associations between illicit and cross-border sources of tobacco and cost of smoking tended to be weaker and did not reach statistical significance in the high social grade. Associations of cigarette and

For peer review only - http://bmjopen.bmj.com/site/about/guidelines.xhtml

RYO consumption with cost tended to be stronger among individuals in the high social grade compared with the low social grade, indicating that they smoke more expensive products.

In Table 4 we tested the interaction between time and cost-minimising strategies to assess whether the influence of these strategies on cost changed over time. The association between smoking cost and factory-made cigarette consumption grew stronger over time. Interactions with RYO consumption, use of illicit and cross-border sources of tobacco did not reach statistical significance.

Results for the sensitivity analyses are presented in the Supplementary Tables. Increasing the proportion of RYO cigarettes within total cigarette consumption was associated with a decrease in cost of smoking of 70.80% when 0% RYO was compared with 100% RYO (95%CI -72.80 to -68.94) (Supplementary Table 1). Note that these results were not corrected for the amount of tobacco used for a RYO cigarette vs a manufactured cigarette. The price per cigarette (Supplementary Table 2) increased with a decrease in factory-made and RYO cigarette consumption. It decreased (around 12%) with the use of either illicit or cross-border sources. The post-hoc analysis, presented in Supplementary Table 3, demonstrated that the results for the analysis performed on data including individuals who reported very high (>£1) or very low (<£0.05) values for price per cigarette would not lead to different conclusions than those from the main analysis.

DISCUSSION

Key findings

Reported cost of smoking in England did not increase over time above the rate of inflation, despite above-inflation tax increases. Factory-made cigarette consumption decreased, while the proportion of RYO and, to a much lesser extent, illicit and cross-border purchases increased. These trends were only evident in lowest social grade. Lowering factory-made cigarette consumption was associated with greater cost reductions than lowering RYO cigarette consumption. Consumption reduction accounted for 59% of variance. Cross-border and illicit tobacco purchases were associated with lower costs, but due to low prevalence, accounted for only 0.2% of variation in cost. Associations were similar across social grades, although illicit sources reduced the cost more strongly in smokers from low social grades than smokers from high social grades.

Limitations

The results of this study should be interpreted in light of the following limitations. Use of illicit and cross-border sources was measured dichotomously over a time frame of six months preceding the interview. This may have biased the results in two ways. First, we lacked information on the frequency of use of these sources. Because any one-time use is counted as using illicit/cross-border sources and the prevalence may therefore not represent, and likely overestimate, the proportion of not full duty-paid purchases out of total tobacco purchases. However, our prevalence rates are comparable with findings from the ITC UK data of 2010/2011 and 2014, in which the source of last purchase was measured.(8, 26) Second, the cost of smoking was measured over an average week, a much smaller timeframe than six months. Associations between smoking cost and tobacco sources may be diluted as a result of non-differential misclassification and the contribution to the cost of smoking may be larger than portrayed. However, given the very small share of 0.2% according to the current analysis, a substantially large share is unlikely.

The data did not contain information on usual brand. Brand switching is a commonly used cost-minimising strategy.(10, 13) Choice of brand may have considerable impact on the cost of smoking,

For peer review only - http://bmjopen.bmj.com/site/about/guidelines.xhtml

due to undershifting; the tobacco industry's strategy to divide tax increases disproportionately among different price segments.(9, 27) In general, undershifting caused low priced brands to have remained cheap, while prices of premium brands have increased.(9, 27, 28) The associations found for the studied cost-minimising strategies may still be confounded by brand switching, as brand switching and the studied strategies are likely to co-occur.(10)

This study covers a period in which tobacco taxes increased above inflation annually, but the cost of smoking did not significantly increase above inflation rates. This may reflect a lack of effect of tax increases on the actual retail price of tobacco due to undershifting.(9, 27-29) The lack of an increasing trend may however also represent selective quitting. Many smokers in England quit during the study period, and smoking prevalence dropped from 19.3% in 2012 to 15.5% in 2016.(19) If smokers who spend more on tobacco are hit harder by a tax increase, they may be more likely to quit in response to increasing tax. The remaining smokers may therefore be those with lower levels of spending to begin with. The current study did not capture any effect of taxes on quitting.

We only collected data on expenditure on smoking, and not expenditure on alternative nicotine products. In England, e-cigarette use increased over the study period.(30) As a fifth of smokers use e-cigarettes,(31) we have underestimated smokers' expenditure on nicotine. Decreases in tobacco consumption may in part have been due to switching to dual-use of combustible cigarettes and e-cigarettes, and the inverse association found between cigarette consumption and smoking cost would have been somewhat weaker if expenditure on e-cigarettes would have been taken into account.

Interpretation

Our results are in line with previous findings that RYO cigarettes are much cheaper than factory-made cigarettes(9) as we found that switching from factory-made to RYO cigarettes is an effective cost-mitigating strategy. In our data, the proportion of RYO use increased over time. This increase has been observed since the early 2000's(29) and appears to continue over time.(8) Proportionally increased use is likely to be a response to an increasing gap in prices between factory-made and RYO.(9) Switching to RYO may have serious public health consequences, as smokers using RYO tobacco are much less sensitive to further price increases.(32) Moreover, smokers from lower social grades are more likely to use RYO tobacco(29), which makes the increasing price gap likely to contribute to growing socioeconomic inequalities in smoking.(12) In order to encourage smoking cessation across social grades, taxes on RYO tobacco need to increase to the same level as factory-made cigarettes, which may be achieved through continued larger increases in RYO tobacco taxes.(8, 17, 33)

We found a small increase in the proportion of smokers who reported purchasing tobacco from illicit or cross-border sources. This is in line with an overview by Rowell et al., showing that the most reliable information on illicit tobacco does not show dramatic increases in use.(34) An analysis of 2002-2014 UK ITC data showed no increases in use of self-reported sources outside the UK or from informal sellers.(8) Illicit trade in cigarettes, measured by the tax gap between consumption and sales of tobacco, decreased from 16% in 2005-2006 to 8% in 2014-2015.(22) However, between 2015-16 and 2016-17, the tax gap increased to 15%,(22) which this paper reflects. The increase in tax gap in recent years seems mainly due to a decline in consumption rather than a growth in illicit trade.(22) The tax gap for RYO tobacco strongly declined from 60% in 2005 to 28% in 2017.(22)

We found that, in line with previous findings,(8, 12, 18) individuals from lower social grades were less likely to purchase tobacco abroad and more likely to use illicit sources, than smokers from higher social grades. Although statistical power was limited, associations between consumption levels and costs tended to be stronger among individuals in the high social grade compared with the low social grade. This may be because smokers in lower social grades are more likely to use lower priced brands,(29)

For peer review only - http://bmjopen.bmj.com/site/about/guidelines.xhtml

for which the reduction of consumption has a smaller effect on the total cost of smoking. The association between illicit sources and the cost of smoking tended to be stronger in smokers from lower social grades, which may be explained by the frequency of using these sources, if smokers from lower social grades use illicit sources on a more regular basis.

Implications

In order to have actual costs of smoking increase above inflation, this study suggests that tax increases during the period of study were not enough to impact weekly tobacco expenditure. Changes in tobacco taxation policy are required, such as sudden larger tax increases, as called for elsewhere,(35) as these would be more impactful. However, tobacco taxation policies need to be designed in a way that takes industry strategies across brand segments and product types into account.(9, 27) As previously called for,(8, 9, 17, 27, 29, 33) this study makes a strong case for continued higher relative tax increases on RYO tobacco products compared with factory-made cigarettes. Other strategies may include maximising specific taxation, strong minimum price policies, plain packaging that removes price promotions from packs (already in effect in the UK), restricting brands to one variant and preventing the introduction of new brands.(9, 27, 36, 37)

Although illicit tobacco formed only a minor threat to the costliness of smoking, a continued increase is undesirable. Action at the national and international level, including effective implementation of the FCTC protocol to eliminate illicit trade, has potential to reduce illicit trade in the near future.(38-40)

Conclusions

At a time when tax increases were designed to raise the cost of tobacco 2-5% above inflation annually, weekly spend on tobacco by smokers in England did not change above inflation. Our study showed that smokers commonly reduce consumption and switch to hand-rolled tobacco, particularly those of more disadvantaged social grades, but that the use of illicit and cross-border sources of tobacco was much less common and hardly contributed to total expenditure on tobacco. Strong future tobacco taxation policies are needed, that take industry strategies across brand segments and product types into account.

For peer review only

For peer review only - http://bmjopen.bmj.com/site/about/guidelines.xhtml

Funding
This work was supported by the National Institute for Health Research Public Health Research (project number 13/43/58). The Smoking Toolkit Study is currently primarily funded by Cancer Research UK (C1417/A22962; C44576/A19501) and has previously also been funded by Pfizer, GSK, and the Department of Health. Department of Health Disclaimer: The views and opinions expressed therein are those of the authors and do not necessarily reflect those of the Public Health Research programme, NIHR, NHS or the Department of Health.
No funders had any involvement in the design of the study, the analysis or interpretation of the data, the writing of the report, or the decision to submit the paper for publication.

Competing interests
RW undertakes consultancy and research for and receives travel funds and hospitality from manufacturers of smoking cessation medications. EB and JB have received unrestricted research funding from Pfizer. MK, TP, AG, and AM have no interests to declare.

Author contributions
JB, AM and RW conceived and designed the study. MAGK prepared and analysed the data, interpreted the findings and drafted the manuscript. AM, TP, JB, RW, EB and AG contributed to the interpretation of the data and provided critical revisions to the manuscript. All authors approved the final version of the paper for submission.

Data sharing statement
For access to the Smoking Toolkit Study please contact Dr Jamie Brown, jamie.brown@ucl.ac.uk.

For peer review only

REFERENCES

1. World Health Organization. WHO report on the gobal tobacco epidemic, 2008: The MPOWER package. Geneva; 2008 2008.
2. Phlips L. Applied Consumption Analysis: Advanced Textbooks in Economics: Elsevier; 2014.
3. Chaloupka FJ, Yurekli A, Fong GT. Tobacco taxes as a tobacco control strategy. Tobacco Control. 2012;21(2):172-80.
4. Biener L, Aseltine Jr RH, Cohen B, Anderka M. Reactions of adult and teenaged smokers to the Massachusetts tobacco tax. American Journal of Public Health. 1998;88(9):1389-91.
5. Brown T, Platt S, Amos A. Equity impact of population-level interventions and policies to reduce smoking in adults: a systematic review. Drug and alcohol dependence. 2014;138:7-16.
6. Hiscock R, Bauld L, Amos A, Platt S. Smoking and socioeconomic status in England: the rise of the never smoker and the disadvantaged smoker. Journal of Public Health. 2012;34(3):390-6.
7. Bosdriesz JR, Willemsen MC, Stronks K, Kunst AE. Socioeconomic inequalities in smoking cessation in 11 European countries from 1987 to 2012. J Epidemiol Community Health. 2015:jech-2014-205171.
8. Partos TR, Gilmore AB, Hitchman SC, Hiscock R, Branston JR, McNeill A. Availability and use of cheap tobacco in the UK 2002-2014: Findings from the International Tobacco Control Project. Nicotine & Tobacco Research. 2017:ntx108.
9. Hiscock R, Branston JR, McNeill A, Hitchman SC, Partos TR, Gilmore AB. Tobacco industry strategies undermine government tax policy: evidence from commercial data. Tobacco Control. 2017:tobaccocontrol-2017-053891.
10. Choi K, Hennrikus D, Forster J, St Claire AW. Use of price-minimizing strategies by smokers and their effects on subsequent smoking behaviors. Nicotine & Tobacco Research. 2012;14(7):864-70.
11. Licht AS, Hyland AJ, O'Connor RJ, Chaloupka FJ, Borland R, Fong GT, et al. How do price minimizing behaviors impact smoking cessation? Findings from the International Tobacco Control (ITC) Four Country Survey. International journal of environmental research and public health. 2011;8(5):1671-91.
12. Licht AS, Hyland AJ, O'Connor RJ, Chaloupka FJ, Borland R, Fong GT, et al. Socio-economic variation in price minimizing behaviors: findings from the International Tobacco Control (ITC) Four Country Survey. International journal of environmental research and public health. 2011;8(1):234-52.
13. White VM, Gilpin EA, White MM, Pierce JP. How do smokers control their cigarette expenditures? Nicotine & tobacco research. 2005;7(4):625-35.
14. Xu X, Pesko MF, Tynan MA, Gerzoff RB, Malarcher AM, Pechacek TF. Cigarette price-minimization strategies by US smokers. American journal of preventive medicine. 2013;44(5):472-6.
15. Pesko MF, Licht AS, Kruger JM. Cigarette price minimization strategies in the United States: price reductions and responsiveness to excise taxes. nicotine & tobacco research. 2013;15(11):1858-66.
16. Pesko MF, Kruger J, Hyland A. Cigarette price minimization strategies used by adults. American journal of public health. 2012;102(9):e19-e21.
17. Young D, Wilson N, Borland R, Edwards R, Weerasekera D. Prevalence, correlates of, and reasons for using roll-your-own tobacco in a high RYO use country: findings from the ITC New Zealand survey. Nicotine Tob Res. 2010;12(11):1089-98.
18. Nagelhout GE, van den Putte B, Allwright S, Mons U, McNeill A, Guignard R, et al. Socioeconomic and country variations in cross-border cigarette purchasing as tobacco tax avoidance strategy. Findings from the ITC Europe Surveys. Tobacco control. 2013:tobaccocontrol-2012-050838.
19. Office for National Statistics. Dataset: Smoking habits in the UK and its constituent countries.; 2018.
20. World Health Organization. WHO report on the global tobacco epidemic 2015: raising taxes on tobacco: World Health Organization; 2015.

For peer review only - http://bmjopen.bmj.com/site/about/guidelines.xhtml

21. Action on Smoking and Health. Timeline of changes in tobacco taxation in the UK from 1993 to the present 2017 [cited 2017. Available from: http://ash.org.uk/information-and-resources/taxation-illicit-trade/taxation/ash-analysis-of-tobacco-tax-increases-in-the-united-kingdom/.
22. HM Revenue & Customs. Measuring tax gaps 2017 edition. Tobacco tax gap estimates for 2016-17.; 2017.
23. Fidler JA, Shahab L, West O, Jarvis MJ, McEwen A, Stapleton JA, et al. 'The smoking toolkit study': a national study of smoking and smoking cessation in England. BMC Public Health. 2011;11(1):1.
24. CPI INDEX 00: ALL ITEMS 2015=100, (2017).
25. Wood SN. Generalized additive models: an introduction with R: Chapman and Hall/CRC; 2017.
26. Guindon GE, Driezen P, Chaloupka FJ, Fong GT. Cigarette tax avoidance and evasion: findings from the International Tobacco Control Policy Evaluation Project. Tobacco control. 2013:tobaccocontrol-2013-051074.
27. Gilmore AB, Tavakoly B, Taylor G, Reed H. Understanding tobacco industry pricing strategy and whether it undermines tobacco tax policy: the example of the UK cigarette market. Addiction. 2013;108(7):1317-26.
28. Marsh L, Cameron C, Quigg R, Hoek J, Doscher C, McGee R, et al. The impact of an increase in excise tax on the retail price of tobacco in New Zealand. Tobacco control. 2015:tobaccocontrol-2015-052259.
29. Gilmore AB, Tavakoly B, Hiscock R, Taylor G. Smoking patterns in Great Britain: the rise of cheap cigarette brands and roll your own (RYO) tobacco. Journal of public health. 2015;37(1):78-88.
30. Beard E, Brown J, Michie S, West R. Is prevalence of e-cigarette and nicotine replacement therapy use among smokers associated with average cigarette consumption in England? A time-series analysis. BMJ open. 2018;8(6):e016046.
31. Brown J, West R, Beard E, Michie S, Shahab L, McNeill A. Prevalence and characteristics of e-cigarette users in Great Britain: findings from a general population survey of smokers. Addictive behaviors. 2014;39(6):1120-5.
32. Tait P, Rutherford P, Saunders C. Do consumers of manufactured cigarettes respond differently to price changes compared with their Roll-Your-Own counterparts? Evidence from New Zealand. Tobacco control. 2013:tobaccocontrol-2013-051010.
33. Young D, Yong H-H, Borland R, Shahab L, Hammond D, Cummings KM, et al. Trends in roll-your-own smoking: findings from the ITC Four-Country Survey (2002–2008). Journal of environmental and public health. 2012;2012.
34. Rowell A, Evans-Reeves K, Gilmore AB. Tobacco industry manipulation of data on and press coverage of the illicit tobacco trade in the UK. Tobacco control. 2014;23(e1):e35-e43.
35. Partos TR, Branston JR, Hiscock R, Gilmore AB, McNeill A. Individualised tobacco affordability in the UK 2002–2014: findings from the International Tobacco Control Policy Evaluation Project. Tobacco control. 2019:tobaccocontrol-2017-054027.
36. Chaloupka FJ. Maximizing the public health impact of alcohol and tobacco taxes. American journal of preventive medicine. 2013;44(5):561-2.
37. Golden SD, Smith MH, Feighery EC, Roeseler A, Rogers T, Ribisl KM. Beyond excise taxes: a systematic review of literature on non-tax policy approaches to raising tobacco product prices. Tobacco Control. 2016;25(4):377-85.
38. McNeill A, Iringe-Koko B, Bains M, Bauld L, Siggens G, Russell A. Countering the demand for, and supply of, illicit tobacco: an assessment of the 'North of England Tackling Illicit Tobacco for Better Health'Programme. Tobacco control. 2013:tobaccocontrol-2013-050957.
39. HM Revenue & Customs. Customs (2015). Tackling illicit tobacco: From leaf to light 2017 [
40. World Health Organization. Protocol to eliminate illicit trade in tobacco products. 2013.

For peer review only - http://bmjopen.bmj.com/site/about/guidelines.xhtml

FIGURES AND TABLES

Table 1: Description of socio-demographics, cost-minimising strategies, and cost of smoking in the overall population of smokers and by social grade.

	Overall population	Social grade		
		Low (N=7,032)	Middle (N=8,138)	High (N=1,797)
Age distribution, % (95%CI)				
18 – 24	18.2 (17.6 to 18.8)	18.7 (17.8 to 19.6)	19.4 (18.6 to 20.3)	11.1 (9.8 to 12.7)
25 – 34	20.8 (20.2 to 21.4)	21.6 (20.6 to 22.5)	20.8 (19.9 to 21.6)	17.6 (15.9 to 19.4)
35 – 44	17.2 (16.7 to 17.8)	16.8 (16.0 to 17.7)	17.2 (16.4 to 18.0)	19.1 (17.4 to 21.0)
45 – 54	17.1 (16.5 to 17.6)	16.1 (15.3 to 17.0)	17.5 (16.6 to 18.3)	18.9 (17.2 to 20.8)
55 – 64	13.9 (13.4 to 14.5)	14.0 (13.2 to 14.8)	13.2 (12.5 to 14.0)	16.7 (15.0 to 18.5)
65+	12.8 (12.3 to 13.3)	12.8 (12.1 to 13.6)	11.9 (11.2 to 12.7)	16.5 (14.9 to 18.3)
Gender, % (95%CI)				
Male	47.5 (46.8 to 48.3)	51.8 (50.6 to 53.0)	44.8 (43.7 to 45.8)	43.2 (40.9 to 45.5)
Female	52.5 (51.7 to 53.2)	48.2 (47.0 to 49.4)	55.2 (54.2 to 56.3)	56.8 (54.5 to 59.1)
Social grade, % (95%CI)				
Low	41.4 (40.7 to 42.2)	-	-	-
Middle	48.0 (47.2 to 48.7)	-	-	-
High	10.6 (10.1 to 11.1)	-	-	-
Factory-made cigarette consumption in cigarettes/week, mean (95%CI)	45.9 (45.1 to 46.7)	43.3 (42.0 to 44.6)	47.1 (45.8 to 48.3)	50.8 (48.3 to 53.4)
RYO cigarette consumption in cigarettes/week, mean (95%CI)	36.2 (35.4 to 37.0)	44.8 (43.4 to 46.2)	31.8 (30.8 to 32.9)	22.0 (20.1 to 24.0)
Use of illicit sources, % (95%CI)	8.2 (7.8 to 8.6)	9.8 (9.1 to 10.5)	7.5 (7.0 to 8.1)	5.0 (4.0 to 6.0)
Cross-border purchase, % (95%CI)	7.4 (7.0 to 7.8)	4.2 (3.8 to 4.7)	8.9 (8.3 to 9.6)	13.2 (11.7 to 14.8)
Cost of smoking in £/week, mean (95%CI)	23.3 (23.0 to 23.5)	22.8 (22.3 to 23.2)	23.5 (23.1 to 23.9)	24.1 (23.2 to 25.0)

Figure 1: Trends in cost of smoking, cigarette consumption, use of illicit sources, and cross-border purchase among smokers in England.

For peer review only

For peer review only - http://bmjopen.bmj.com/site/about/guidelines.xhtml

Table 2: Percentage difference in cost of smoking for socio-demographics and cost-minimising strategies from Generalised Additive Models (GAM).

	Percentage difference in cost of smoking (100β with 95%CI)		
	**Model 1** Baseline model	**Models 2 to 5** Adjusted for socio-demographics	**Model 6** All variables
Age			
Per 10 years increase	6.68 (5.94 to 7.43)		0.13 (-0.40 to 0.66)
Gender			
Male	ref		ref
Female	0.78 (-1.75 to 3.30)		0.11 (-1.67 to 1.90)
Social grade			
Low	ref		ref
Middle	-0.99 (-3.66 to 1.68)		1.44 (-0.45 to 3.34)
High	-6.93 (-11.25 to -2.59)		-0.91 (-4.00 to 2.17)
Factory-made cigarette consumption[a]			
Per 10 cigarettes decrease		-9.95 (-10.12 to -9.77)	-12.65 (-12.84 to -12.46)
RYO cigarette consumption[b]			
Per 10 cigarettes decrease		1.15 (0.91 to 1.39)	-5.58 (-5.77 to -5.38)
Use of illicit sources[c]			
No use of illicit sources in last 6 months		ref	ref
Used illicit sources in last 6 months		-7.23 (-11.84 to -2.62)	-10.17 (-13.46 to -6.88)
Cross-border purchase[d]			
No cross-border purchase in last 6 months		ref	ref
Cross-border purchase in last 6 months		-9.48 (-14.31 to -4.65)	-10.64 (-14.04 to -7.25)

RYO = roll-your-own

[a] 49.7% of variance in spending accounted for by factory-made cigarette consumption.

[b] 8.8% of variance in spending accounted for by RYO cigarette consumption.

[c] 0.1% of variance in spending accounted for by use of illicit sources of tobacco.

[d] 0.1% of variance in spending accounted for by use of cross-border sources of tobacco.

For peer review only

For peer review only - http://bmjopen.bmj.com/site/about/guidelines.xhtml

Table 3: Percentage difference in cost of smoking for socio-demographics and cost-minimising strategies from Generalised Additive Models (GAM), stratified by social grade.

	Social grade				
	Low (N=7,032)	Middle (N=8,138)		High (N=1,797)	
	**100β with 95%CI**	**100β with 95%CI**	**P-value for interaction, middle vs low**	**100β with 95%CI**	**P-value for interaction, high vs low**
Age					
Per 10 years increase	-0.18 (-0.95 to 0.59)	-0.14 (-0.65 to 0.92)		0.35 (-1.46 to 2.16)	
Gender					
Male	ref	ref		ref	
Female	0.65 (1.99 to 3.29)	-0.37 (-2.98 to 2.24)		-1.09 (-7.00 to 4.82)	
Factory-made cigarette consumption					
Per 10 cigarettes decrease	-11.36 (-11.64 to -11.08)	-13.19 (-13.47 to -12.92)	<0.001	-15.19 (-15.81 to -14.58)	<0.001
RYO cigarette consumption					
Per 10 cigarettes decrease	-4.73 (-4.99 to -4.47)	-6.07 (-6.37 to -5.76)	0.010	-7.74 (-8.54 to -6.95)	0.011
Use of illicit sources					
No use of illicit sources in last 6 months	ref	ref		ref	
Used illicit sources in last 6 months	-12.57 (-17.07 to -8.07)	-7.47 (-12.45 to -2.49)	0.027	-2.94 (-16.51 to 10.64)	0.059
Cross-border purchase					
No cross-border purchase in last 6 months	ref	ref		ref	
Cross-border purchase in last 6 months	-14.71 (-21.24 to -8.18)	-10.79 (-15.33 to -6.25)	0.194	-7.54 (-16.17 to 1.10)	0.035

RYO = roll-your-own
Note: All models were adjusted for all variables in the table.

Table 4: Increase per year in the % difference in cost of smoking for cost-minimising strategies from Generalised Additive Models (GAM)

	**Increase in the % difference in cost of smoking for each consecutive year (95%CI)**	**P-value for interaction**
Factory-made cigarette consumption · time	-0.15 (-0.26 to 0.03)	0.012
RYO cigarette consumption · time	0.02 (-0.10 to 0.14)	0.740
Illicit sources · time	1.02 (-1.36 to 3.40)	0.402
Cross-border purchase · time	-0.14 (-2.55 to 2.26)	0.909

RYO = roll-your-own tobacco
Note: Models were adjusted for age, gender, social grade, and other cost-minimising strategies in the table.

For peer review only - http://bmjopen.bmj.com/site/about/guidelines.xhtml

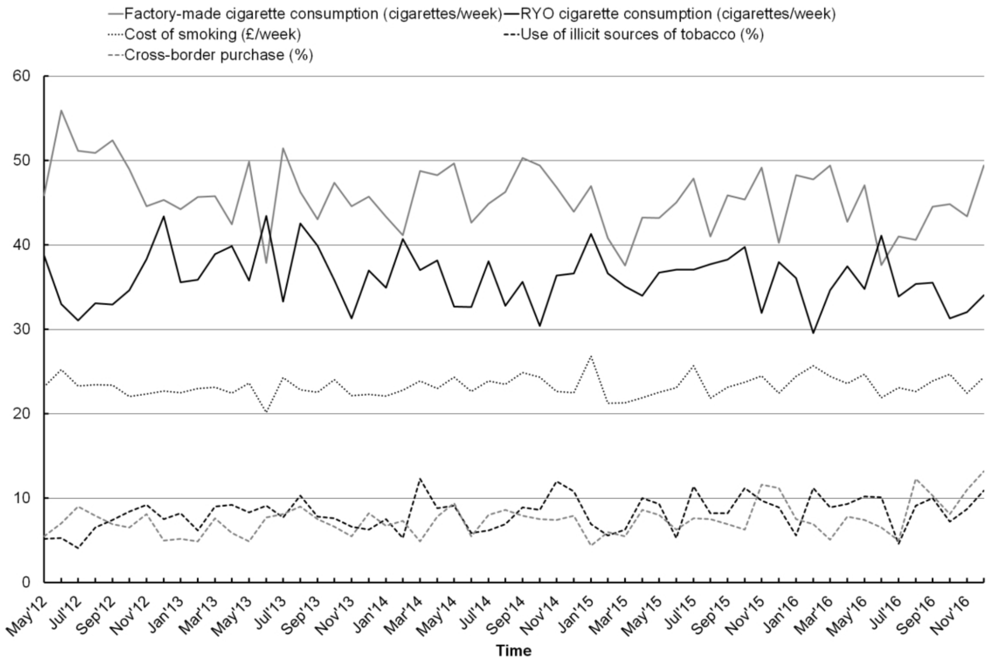

Trends in cost of smoking, cigarette consumption, use of illicit sources, and cross-border purchase among smokers in England.

198x132mm (300 x 300 DPI)

SUPPLEMENTARY FILE

Mirte AG Kuipers, Timea R Partos, Jamie Brown, Emma Beard, Anna Gilmore5, Robert West, Ann McNeill
Smokers' strategies across social grades to minimise the cost of smoking in a period with annual tax increases: Evidence from a national survey in England
BMJ Open

Supplementary Table 1: Results from Generalised Additive Models (GAM) for cost of smoking, with total cigarette consumption and proportion of RYO use.

	**Percentage difference in cost of smoking (100β with 95%CI)**
	**Model 6** Adjusted for other cost-minimising strategies
Age (per 10 years increase)	-0.27 (-0.79 to 0.26)
Gender	
Male	ref
Female	1.22 (-0.56 to 2.99)
Social grade	
Low	ref
Middle	2.48 (0.60 to 4.37)
High	-0.76 (-3.83 to 2.30)
Total cigarette consumption (per 10 cigarettes decrease)	-9.62 (-9.78 to -9.45)
Proportion of RYO cigarette consumption[a]	-70.80 (-72.80 to -68.94)
Use of illicit sources	-10.48 (-13.75 to -7.21)
Use of cross-border sources	-9.65 (-13.03 to -6.27)

[a] Represents the difference between 0% RYO and 100% RYO cigarettes of total consumption.

Supplementary Table 2: Results from Generalised Additive Models (GAM) for price per cigarette.

	**Percentage difference in price per cigarette (100β with 95%CI)**
	**Model 6** Adjusted for other cost-minimising strategies
Age (per 10 years increase)	-0.80 (-1.25 to -0.34)
Gender	
Male	ref
Female	1.59 (0.06 to 3.11)
Social grade	
Low	ref
Middle	5.73 (4.11 to 7.35)
High	10.42 (7.78 to 13.06)
Factory-made cigarette consumption (per 10 cigarettes decrease)	0.77 (0.61 to 0.93)
RYO cigarette consumption (per 10 cigarettes decrease)	7.62 (7.45 to 7.78)
Use of illicit sources	-12.29 (-15.11 to -9.48)
Use of cross-border sources	-12.19 (-15.10 to -9.28)

For peer review only

Supplementary Table 3: Results from Generalised Additive Models (GAM) for cost of smoking, in the population from which respondents were not excluded based on the reported value for price/cigarette (N=17,789).

	**Percentage difference in cost of smoking (100β with 95%CI)**
	**Model 6**<br>Adjusted for other cost-minimising strategies
Age (per 10 years increase)	0.36 (-0.21 to 0.94)
Gender	
Male	ref
Female	1.32 (-0.61 to 3.25)
Social grade	
Low	ref
Middle	0.94 (-1.11 to 2.99)
High	-1.40 (-4.74 to 1.94)
Factory-made cigarette consumption[a]	
Per 10 cigarettes decrease	-11.95 (-11.74 to -12.15)
RYO cigarette consumption[b]	
Per 10 cigarettes decrease	-4.31 (-4.11 to -4.51)
Use of illicit sources[c]	
No use of illicit sources in last 6 months	ref
Used illicit sources in last 6 months	-9.85 (-13.37 to -6.33)
Cross-border purchase[d]	
No cross-border purchase in last 6 months	ref
Cross-border purchase in last 6 months	-16.51 (-20.15 to -12.88)

RYO = roll-your-own

For peer review only

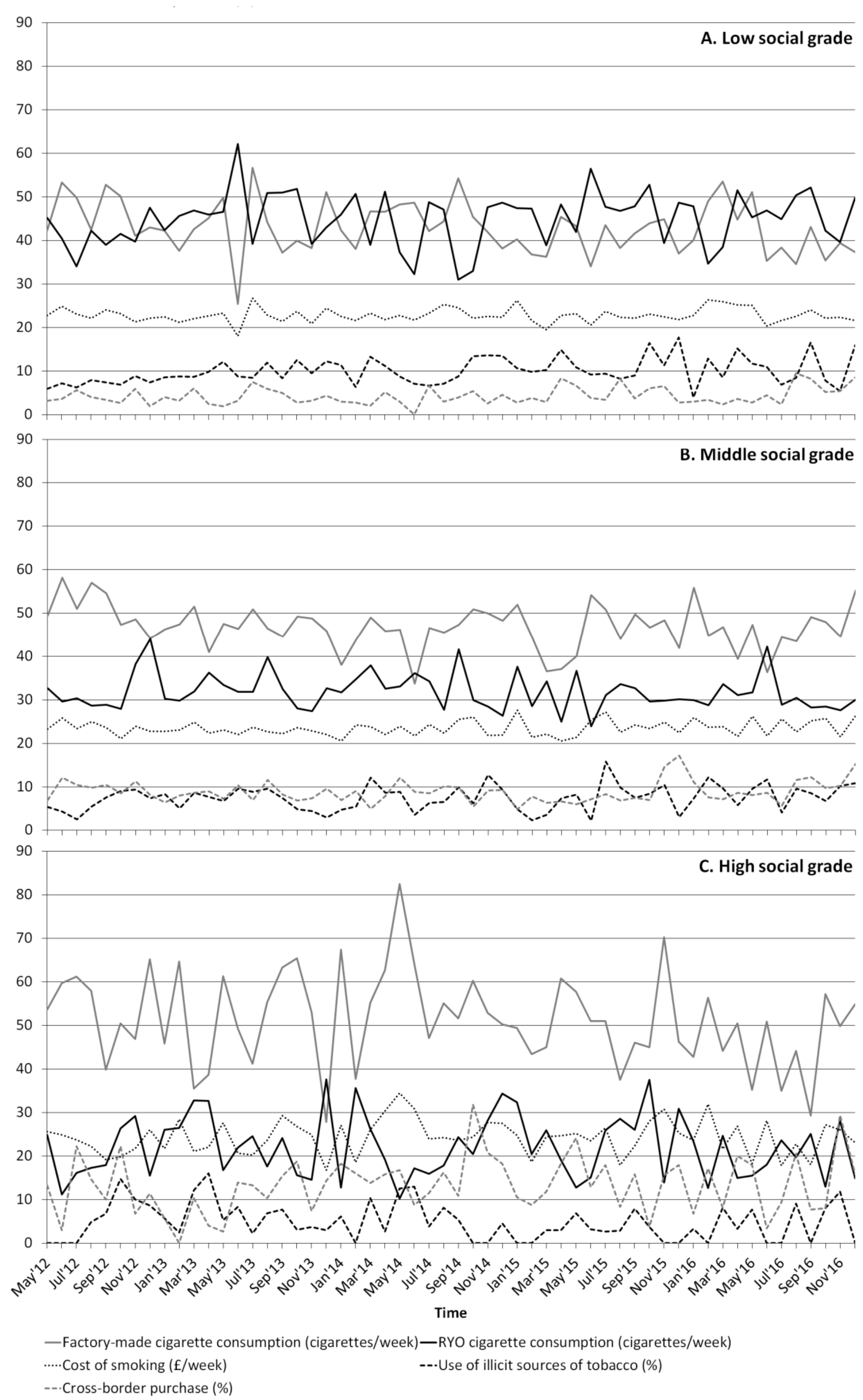

Supplementary Figure 1: Trends in cost of smoking, cigarette consumption, use of illicit sources, and cross-border purchase among smokers in England, stratified by social grade.

STROBE Statement—Checklist of items that should be included in reports of ***cross-sectional studies***

	Page no.	Item No	Recommendation
**Title and abstract**	2	1	(*a*) Indicate the study's design with a commonly used term in the title or the abstract
	2		(*b*) Provide in the abstract an informative and balanced summary of what was done and what was found
	**Introduction**		
Background/rationale	3	2	Explain the scientific background and rationale for the investigation being reported
Objectives	3	3	State specific objectives, including any prespecified hypotheses
	**Methods**		
Study design	4	4	Present key elements of study design early in the paper
Setting	4	5	Describe the setting, locations, and relevant dates, including periods of recruitment, exposure, follow-up, and data collection
Participants	4	6	(*a*) Give the eligibility criteria, and the sources and methods of selection of participants
Variables	4,5	7	Clearly define all outcomes, exposures, predictors, potential confounders, and effect modifiers. Give diagnostic criteria, if applicable
Data sources/ measurement	4	8*	For each variable of interest, give sources of data and details of methods of assessment (measurement). Describe comparability of assessment methods if there is more than one group
Bias	5	9	Describe any efforts to address potential sources of bias
Study size	4	10	Explain how the study size was arrived at
Quantitative variables	4,5	11	Explain how quantitative variables were handled in the analyses. If applicable, describe which groupings were chosen and why
Statistical methods	5	12	(*a*) Describe all statistical methods, including those used to control for confounding
	5		(*b*) Describe any methods used to examine subgroups and interactions
	4		(*c*) Explain how missing data were addressed
	N/A		(*d*) If applicable, describe analytical methods taking account of sampling strategy
	5		(*e*) Describe any sensitivity analyses
	**Results**		
Participants	N/A	13*	(a) Report numbers of individuals at each stage of study—eg numbers potentially eligible, examined for eligibility, confirmed eligible, included in the study, completing follow-up, and analysed
	N/A		(b) Give reasons for non-participation at each stage
	N/A		(c) Consider use of a flow diagram
Descriptive data	5,12	14*	(a) Give characteristics of study participants (eg demographic, clinical, social) and information on exposures and potential confounders
	4		(b) Indicate number of participants with missing data for each variable of interest
Outcome data	fig1	15*	Report numbers of outcome events or summary measures
Main results	6,13	16	(*a*) Give unadjusted estimates and, if applicable, confounder-adjusted

For peer review only

For peer review only - http://bmjopen.bmj.com/site/about/guidelines.xhtml

			estimates and their precision (eg, 95% confidence interval). Make clear which confounders were adjusted for and why they were included
	N/A		(*b*) Report category boundaries when continuous variables were categorized
	N/A		(*c*) If relevant, consider translating estimates of relative risk into absolute risk for a meaningful time period
Other analyses	6, Suppl. tables	17	Report other analyses done—eg analyses of subgroups and interactions, and sensitivity analyses
**Discussion**			
Key results	6,7	18	Summarise key results with reference to study objectives
Limitations	7	19	Discuss limitations of the study, taking into account sources of potential bias or imprecision. Discuss both direction and magnitude of any potential bias
Interpretation	7,8	20	Give a cautious overall interpretation of results considering objectives, limitations, multiplicity of analyses, results from similar studies, and other relevant evidence
Generalisability	8	21	Discuss the generalisability (external validity) of the study results
**Other information**			
Funding	9	22	Give the source of funding and the role of the funders for the present study and, if applicable, for the original study on which the present article is based

*Give information separately for exposed and unexposed groups.

Note: An Explanation and Elaboration article discusses each checklist item and gives methodological background and published examples of transparent reporting. The STROBE checklist is best used in conjunction with this article (freely available on the Web sites of PLoS Medicine at http://www.plosmedicine.org/, Annals of Internal Medicine at http://www.annals.org/, and Epidemiology at http://www.epidem.com/). Information on the STROBE Initiative is available at www.strobe-statement.org.

For peer review only

BMJ Open

Smokers' strategies across social grades to minimise the cost of smoking in a period with annual tax increases: Evidence from a national survey in England

Journal:	*BMJ Open*
Manuscript ID	bmjopen-2018-026320.R2
Article Type:	Research
Date Submitted by the Author:	15-Apr-2019
Complete List of Authors:	Kuipers, Mirte; Academic Medical Center, University of Amsterdam, Partos, Timea; Kings College London, Addictions McNeill, Ann; King's College London, UK Centre for Tobacco Control Studies, National Addiction Centre, Institute of Psychiatry Beard, Emma; UCL, Gilmore, Anna; Tobacco Control Research Group, UK Centre for Tobacco and Alcohol Studies, Department for Health, University of Bath West, Robert; University College London Brown, Jamie; University College London, Psychology & Language Sciences
**Primary Subject Heading**:	Smoking and tobacco
Secondary Subject Heading:	Epidemiology, Public health
Keywords:	EPIDEMIOLOGY, PREVENTIVE MEDICINE, PUBLIC HEALTH

SCHOLARONE™
Manuscripts

Smokers' strategies across social grades to minimise the cost of smoking in a period with annual tax increases: Evidence from a national survey in England

Mirte AG Kuipers, PhD, corresponding author
Department of Epidemiology and Public Health, Health Behaviour Research Centre, University College London, London, UK
Department of Public Health, Amsterdam Public Health Research Institute, Academic Medical Center – University of Amsterdam, P.O. Box 22660, 1100 DD, Amsterdam, The Netherlands
E-mail address: m.a.kuipers@amc.uva.nl

Timea R Partos, PhD
Department of Addictions, Institute of Psychiatry, Psychology and Neuroscience, King's College London, 4 Windsor Walk, Denmark Hill, London, SE5 8BB, UK

Ann McNeill, PhD
Department of Addictions, Institute of Psychiatry, Psychology and Neuroscience, King's College London, 4 Windsor Walk, Denmark Hill, London, SE5 8BB, UK

Emma Beard, PhD
Research Department of Clinical, Educational and Health Psychology, University College London, 1-19 Torrington Place, London, WC1E 7HB, UK

Anna Gilmore, PhD
Tobacco Control Research Group, Department for Health, University of Bath, Claverton Down, Bath, BA2 7AY, UK

Robert West, PhD
Department of Epidemiology and Public Health, Health Behaviour Research Centre, University College London, 1-19 Torrington Place, London, WC1E 7HB, UK

Jamie Brown, PhD
Research Department of Clinical, Educational and Health Psychology, University College London, 1-19 Torrington Place, London, WC1E 7HB, UK

Word Count: 3,935
Abstract: 275

For peer review only

ABSTRACT

Objectives: To assess associations between smokers' strategies to minimise how much their smoking costs and cost of smoking among smokers across three social grades during a period of annual tax increases in England.
Design: Repeat cross-sectional.
Setting: England, May 2012-Dec 2016.
Participants: 16,967 adult smokers in 56 monthly surveys with nationally representative samples.
Measures and analysis: Weighted generalised additive models (GAM) assessed associations between four cost-minimising strategies (factory-made and roll-your-own (RYO) cigarette consumption levels, illicit and cross-border purchases) and cost of smoking (£/week). We adjusted for inflation rate, age, gender, and secular and seasonal trends.
Results Cost of smoking did not increase above the rate of inflation. Factory-made cigarette consumption decreased, while proportion of RYO and, to a much lesser extent, illicit and cross-border purchases increased. These trends were only evident in lowest social grade. Cost of smoking was 12.99% lower with consumption of 10 fewer factory-made cigarettes (95%CI=-13.18;-12.80), and 5.86% lower with consumption of 10 fewer RYO cigarettes (95%CI=-5.66;-6.06). Consumption levels accounted for 60% of variance in cost. Cross-border and illicit tobacco purchases were associated with 9.64% (95%CI=-12.94;-6.33) and 9.47% (95%CI=-12.74;-6.20) lower costs, respectively, but due to low prevalence, accounted for only 0.2% of variation. Associations were similar across social grades, although weaker for illicit and cross-border purchases and stronger for consumption in higher social grades compared with lower social grades.
Conclusion During a period of annual tax increases, the weekly cost of smoking did not increase above inflation. Cost-minimising strategies increased, especially among more disadvantaged smokers. Reducing cigarette consumption and switching to roll-your-own tobacco explained a large part of cost variation, while use of illicit and cross-border purchasing played only a minor role.

Keywords: Smoking, costs, spending, expenditure, tobacco consumption, roll-your-own tobacco, illicit tobacco, cross-border purchasing

STRENGTHS AND LIMITATIONS OF THIS STUDY

Strengths

- This study used monthly data on a 4.5 year period from a large nationally representative sample of smokers across social grades.
- Data allowed comparison of the contribution of multiple cost minimising strategies to the cost of smoking.

Limitations

- Illicit and cross-border purchases were measured as any purchases over the past six months, and the variables may therefore not reflect the frequency of the use of these sources.
- The data did not contain information on usual brand of cigarettes, and we therefore did not take brand switching into account.
- We cannot rule out selective quitting, which may have affected the observed trend in cost of smoking if smokers who spend less are be more likely to quit than those who spend more.

For peer review only - http://bmjopen.bmj.com/site/about/guidelines.xhtml

INTRODUCTION

Increasing taxes on tobacco is considered among the most effective ways of reducing smoking prevalence.(1) In line with economic theory,(2) the resulting increased costs of smoking may provoke quit attempts, reduce smoking consumption, and deter uptake.(3, 4) Notably, tax increases are among the few interventions to show greater effectiveness in lower, compared to higher, socioeconomic strata,(5) which is important given the association between smoking and disadvantage in England(6) and other countries with a mature smoking epidemic.(7) The effectiveness of tax increases may however be diminished by the increased availability of cheap tobacco(8, 9) and cost-minimising strategies that smokers develop in response.(10, 11)

Studies across high-income countries estimate that between half and three quarters of all smokers apply cost-minimising strategies(10-14) such as cutting down cigarette consumption, switching to a lower priced brand, switching to roll-your-own (RYO) tobacco, or evading or avoiding tobacco taxation by changing the source of purchase to illicit or cross-border/duty free sources, respectively (collectively known as non-UK duty-paid).(8, 10-13) US smokers who use one or more cost-minimising strategy have been shown to significantly reduce their costs, on average by 22%.(14, 15) Moreover, the use of cost-minimising strategies shows social patterning(16) with smokers of lower socioeconomic status (SES) being more likely to smoke RYO cigarettes and use cheaper factory-made cigarette brands,(12, 17) and smokers of higher SES being more likely to purchase untaxed tobacco products.(12, 18) The relative importance of different strategies however remains to be quantified, as well as the extent to which strategies differentially affect the cost of smoking among different socioeconomic groups.

The current study was set in the UK, where there has been a marked decrease in smoking prevalence (from 19.3% in 2012 to 15.5% in 2016(19)) and where some use of cost-minimising strategies has been demonstrated using data from the International Tobacco Control policy evaluation project.(8, 11, 12, 18) The UK has among the highest tobacco tax rates worldwide.(20) In 2012-2016, taxes on all tobacco products increased nationwide in March of each year, by 5% above inflation in 2012, and 2% above inflation in 2013-2015. In 2016, a differential tax of 5% above inflation for RYO tobacco and 2% above inflation for factory-made cigarettes was applied.(21) Between 2002 and 2014, the proportion of UK smokers who used at least some RYO tobacco increased substantially, from 30 to 45%.(8) With RYO prices (per stick) being less than half of those of factory-made cigarettes,(8, 9) switching to RYO seems effective in lowering the cost of smoking. Between 2002 and 2014, use of tobacco from non-UK duty-paid sources remained consistent or even decreased,(8, 22) but increased slightly after 2015.(22) The extent to which the use of non-UK duty-paid tobacco contributes to mitigating the costs of smoking, and its relative importance compared with RYO tobacco, has not been previously established, especially across different social grade levels.

This study examined the extent to which the use of cost-minimising strategies allowed smokers from different social grades in England to minimise the actual cost of smoking in 2012-2016. We used data from the Smoking Toolkit Study (STS), which is a repeated cross-sectional monthly survey of the English population. The use of the STS allowed for the study of much more detailed trends and comprehensive measures of illicit tobacco use, in recent nationally representative data.
Specifically, the aims of this study were:

1. to describe trends in the cost of smoking (smokers' self-reported weekly spend on tobacco) and use of cost-minimising strategies between 2012 and 2016 overall and by social grade;
2. to assess in the general smoking population and across social grades, the association of cost of smoking with:
    a. the number of cigarettes smoked per week, both factory-made and RYO;

For peer review only - http://bmjopen.bmj.com/site/about/guidelines.xhtml

b. and the purchase of non-UK duty-paid tobacco by means of purchase from either illicit or cross-border sources.

METHODS

Data and study population

Data were collected as part of the ongoing STS, a national repeated cross-sectional survey of tobacco use in the general population of England. Each month, a new sample of approximately 1700 adults aged ≥16 years is selected using a form of random location sampling. Individuals complete a face-to-face computer-assisted household interview survey with a trained interviewer. The STS samples have been shown to be nationally representative in their sociodemographic composition and proportion of smokers. Full details of the STS methods have been described elsewhere.(23) Ethical approval was granted by the University College London ethics committee.

We used data from 56 monthly waves from May 2012 to December 2016. May 2012 was selected as a starting point, as information on source of tobacco purchase was first measured from this wave. Out of a total of 97,074 respondents, we excluded non-smokers (N=78,184) and respondents with missing smoking status (N=68). Of 18,822 smokers, respondents with missing information on age (N=66) and respondents under 18 years of age (N=259) were excluded, because they could not legally purchase tobacco. We excluded respondents with implausible values for cost of smoking (N=837, assumptions for plausible spending are described below), smokers who did not report their weekly spend on tobacco (N=455), and smokers who did not report their cigarette consumption, or reported it to be zero (N=319). We included 16,967 current daily and non-daily cigarette smokers.

Patient and public involvement

This study involved secondary data analysis of existing data from the Smoking Toolkit Study (STS) surveys. Participants and public were not involved in the current study.

Measurements

The cost of smoking was measured as self-reported weekly spending (in £) on tobacco. Respondents were asked the following open-ended question: "On average about how much per week do you think you spend on cigarettes or tobacco?". The cost of smoking was adjusted for inflation using Consumer Prices Index (CPI) data of all items from the Office for National Statistics (24), with December 2016 as the reference. Only smokers who adhered to three liberal assumptions of plausible levels of consumption and expenditure per week were included in the analysis, which led to the exclusion of 4.7% of smokers (N=837). The three assumptions included: 1) smokers smoke a maximum of 560 cigarettes per week (N=8), 2) spending does not exceed 280 pounds per week (N=7), and 3) single cigarettes cost between £0.05 and £1 (N=830).

Cost-minimising strategies included 1) reducing consumption of factory-made, 2) reducing consumption of RYO cigarettes (i.e. a cheap alternative for factory-made cigarettes(9)), non-UK duty paid tobacco from 3) illicit sources, and 4) cross border sources. Factory-made and RYO cigarette consumption were treated as separate continuous variables in all analyses and were expressed in cigarettes per week. Respondents estimated for both factory-made and RYO cigarettes how many cigarettes they smoked per week. For exclusive factory-made cigarette users, RYO consumption levels were zero, and for exclusive RYO users, factory-made cigarette consumption levels were zero.

For peer review only - http://bmjopen.bmj.com/site/about/guidelines.xhtml
For peer review only

Purchase from illicit sources was measured as self-reported use of any of the following sources of tobacco at least once in the last 6 months: under the counter (from newsagent, off-license, or corner shop), pub (somebody comes around selling cheap), people who sell cheap cigarettes on the street, people in the local area who are a trusted source of cheap cigarettes, or cheap from friends. Cross-border purchasing was measured as self-reported use of cigarettes purchased abroad at least once in the last 6 months. Both were measured as dichotomous variables. Duty free sources within the UK were not specified as a response option and some respondents may have included these in their definition of cross-border sources.

Sociodemographic characteristics measured were gender, age and social grade. Social grade was assigned by the interviewer based on the occupation of the chief income earner of the household and used the National Readership Survey classification system to distinguish three categories: *low*: non-working class and (manual) working class (National Readership Survey (NRS) social grades D and E), *middle*: skilled working class and lower middle class (NRS grade C), and *high*: middle class and upper middle class (NRS grades A and B).

Time was measured in months throughout the study period. To control for seasonality (month-of-year effects), the month within the year ('calendar month') was coded as January=1 to December=12.

Statistical analysis

Data were analysed in R V.3.3.2. The analysis plan was registered on the Open Science Framework prior to data analysis (https://osf.io/ju6tf/). All data and analyses were weighted based on gender, working status, prevalence of children in the household, age, social grade and region, see Fidler et al.(23) The use of weighted data was not reported in the analysis plan, but was later decided on to improve the generalisability of the results to the general population of England.

Descriptive statistics are given for the overall sample and stratified by social grade. Trends are graphically described and linear trends were tested using univariate generalised linear models.

Generalised additive models (GAMs) were used to assess the association between cost-minimising strategies and cost of smoking. GAMs are a type of generalised linear model that allow more sophisticated control for non-linear processes, in this case secular and seasonal trends, than standard linear regression models.(25) The natural log of smoking cost was used, in order to achieve a normal distribution. Results are presented as 100·β, which in this log-level model can be interpreted as the expected % difference in cost of smoking for a unit increase in the covariate. All models included cyclic cubic regression splines for time (maximum of 5 knots; one for each year) and month within the year (maximum of 12 knots; one for each month). There was no evidence of autocorrelation between time periods according to the autocorrelation function (ACF) and partial ACF, and both the Durbin-Watson test and Breusch-Godfrey test were not statistically significant (p-values respectively 0.26 and 0.42).

Model 1 included socio-demographic characteristics (age, gender, and social grade). Models 2 to 5 included each cost-minimising strategy separately, adjusting for socio-demographic characteristics. The fully adjusted model (Model 6) included all cost-minimising strategies plus socio-demographics. Model 6 was stratified according to social grade. We assessed effect modification by social grade, by testing interaction between social grade and cost-minimising strategies. Interaction was also tested between cost-minimising strategies and time, in order to assess whether the influence of these strategies on cost changed over time.

We performed two sensitivity analyses using Model 6. First, factory-made cigarette and RYO cigarette consumption were replaced by total cigarette consumption and RYO proportion of that total, as an

For peer review only - http://bmjopen.bmj.com/site/about/guidelines.xhtml

alternative way of measuring the use of RYO as a substitute for factory-made cigarettes and reflecting its relative cost. Second, the cost per cigarette was used as the outcome instead of cost of smoking per week, as an alternative way of measuring the cost of smoking, that is less dependent on the level of consumption. A post-hoc analysis of Model 6 was carried out in the dataset in which we did not exclude values of cost of smoking based on single cigarettes cost (N=17,789).

RESULTS

Table 1 presents the description of the study population. Factory-made cigarette consumption was lower in the low social grade, while the consumption of RYO tobacco was higher. Illicit sources were more often used in the low social grade, while cross-border purchases were more common in the high social grade. For smokers in the middle social grade, figures for all four price minimising strategies and for reported cost of smoking were in between those of smokers in the low and high social grade.

Figure 1 shows the trends in cost of smoking, consumption, and use of illicit and cross-border sources of tobacco. No significant linear trend was found in the cost of smoking (increase of £0.09 per year, 95%CI-0.16 to 0.33, p=0.486). The number of RYO cigarettes consumed per week did not significantly change over time (0.32 cigarettes per year, 95%CI -0.36 to 1.00, p=0.346), but there was a significant linear decreasing trend in factory-made cigarette consumption (-1.53 cigarettes per year, 95%CI -2.28 to -0.79, p<0.001). This means that within total cigarette consumption (i.e., the sum of RYO and factory-made cigarettes), the proportion of RYO consumption increased (+1.36% per year, 95%CI 0.69 to 2.03, p<0.001, data not shown in Figure 1). We found increasing trends in use of illicit sources (+0.53% per year, 95%CI 0.14 to 0.92, p=0.008) and cross-border purchase (+0.41% per year, 95%CI -0.12 to 0.83, p=0.056). A post-hoc analysis showed that cross-border purchasing mostly increased in the second half of 2016, but much less up to July 2016 (+0.10 per year, 95%CI -0.36 to 0.55, p=0.664). Trends by social grade are presented in Supplementary Figure 1. Linear tests showed that the trends observed in the total population were stronger, and only significant among smokers in the low social grade.

Table 2 presents the associations between cost-minimising strategies and smoking cost. Cost of smoking was higher with increasing age, and higher in the low social grade than in the high social grade. In the fully adjusted model (Model 6), the spline terms for trends over the years (p=0.016) and months (p=0.036) were significant (not presented in table). Model 6 shows that most of the difference between the high and low social grades were attenuated by consumption or source of purchase. When controlling for consumption of factory-made cigarettes in Model 6, a decrease of ten RYO cigarettes per week was associated with 5.86% lower costs of smoking (95%CI:-5.66;-6.06). Controlling for RYO consumption, a decrease in consumption of ten factory-made cigarettes was associated with a 12.99% decrease in costs (95%CI:-12.80;-13.18). Both the use of illicit and cross-border sources of tobacco reduced the cost of smoking (Illicit: -9.64%, 95%CI: -12.94 to -6.33; cross-border: -9.47%, 95%CI: -12.74 to -6.20). Due to their low prevalence, use of illicit and cross-border sources combined accounted for only 0.2% of variation in cost of smoking, while factory-made cigarette consumption and RYO consumption accounted for 50% and 10% of the variation in cost of smoking, respectively.

Table 3 shows the associations between cost-minimising strategies and the cost of smoking, by social grade. The same patterns were found in all three groups of social grade, with decreased consumption levels, and use of illicit and cross-border sources all associated with lower cost of smoking although associations between illicit and cross-border sources of tobacco and cost of smoking tended to be weaker and did not reach statistical significance in the high social grade. Associations of cigarette and RYO consumption with cost tended to be stronger among individuals in the high social grade compared with the low social grade, indicating that they smoke more expensive products.

For peer review only - http://bmjopen.bmj.com/site/about/guidelines.xhtml

In Table 4 we tested the interaction between time and cost-minimising strategies to assess whether the influence of these strategies on cost changed over time. The association between smoking cost and factory-made cigarette consumption tended to grow stronger over time with -0.12 percentage points per year (95%CI:-0.23 to 0.00). Interactions with RYO consumption, use of illicit and cross-border sources of tobacco did not reach statistical significance.

Results for the sensitivity analyses are presented in the Supplementary Tables. Increasing the proportion of RYO cigarettes within total cigarette consumption (Supplementary Table 1) was associated with a decrease in cost of smoking of 70.41% when 0% RYO was compared with 100% RYO (95%CI -72.29 to -68.53). Note that these results were not corrected for the amount of tobacco used for a RYO cigarette vs a manufactured cigarette. The cost per cigarette (Supplementary Table 2) increased with a decrease in factory-made and RYO cigarette consumption. It decreased (around 12%) with the use of either illicit or cross-border sources. The post-hoc analysis, presented in Supplementary Table 3, demonstrated that the results for the analysis performed on data including individuals who reported very high (>£1) or very low (<£0.05) values for cost per cigarette would not lead to different conclusions than those from the main analysis.

DISCUSSION

Key findings

Reported cost of smoking in England did not increase over time above the rate of inflation, despite above-inflation tax increases. Factory-made cigarette consumption decreased, while the proportion of RYO and, to a much lesser extent, illicit and cross-border purchases increased. These trends were only evident in lowest social grade. Lowering factory-made cigarette consumption was associated with greater cost reductions than lowering RYO cigarette consumption. Consumption reduction accounted for 60% of variance. Cross-border and illicit tobacco purchases were associated with lower costs, but due to low prevalence, accounted for only 0.2% of variation in cost. Associations were similar across social grades, although illicit sources reduced the cost more strongly in smokers from low social grades than smokers from high social grades.

Limitations

The results of this study should be interpreted in light of the following limitations. Use of illicit and cross-border sources was measured dichotomously over a time frame of six months preceding the interview. This may have biased the results in two ways. First, we lacked information on the frequency of use of these sources. Because any one-time use is counted as using illicit/cross-border sources and the prevalence may therefore not represent, and likely overestimate, the proportion of not full duty-paid purchases out of total tobacco purchases. However, our prevalence rates are comparable with findings from the ITC UK data of 2010/2011 and 2014, in which the source of last purchase was measured.(8, 26) Second, the cost of smoking was measured over an average week, a much smaller timeframe than six months. Associations between smoking cost and tobacco sources may be diluted as a result of non-differential misclassification and the contribution to the cost of smoking may be larger than portrayed. However, given the very small share of 0.2% according to the current analysis, a substantially large share is unlikely.

The data did not contain information on usual brand. Brand switching is a commonly used cost-minimising strategy.(10, 13) Choice of brand may have considerable impact on the cost of smoking, due to undershifting; the tobacco industry's strategy to divide tax increases disproportionately among

For peer review only - http://bmjopen.bmj.com/site/about/guidelines.xhtml

different price segments.(9, 27) In general, undershifting caused low priced brands to have remained cheap, while prices of premium brands have increased.(9, 27, 28) The associations found for the studied cost-minimising strategies may still be confounded by brand switching, as brand switching and the studied strategies are likely to co-occur.(10)

This study covers a period in which tobacco taxes increased above inflation annually, but the cost of smoking did not significantly increase above inflation rates. This may reflect a lack of effect of tax increases on the actual retail price of tobacco due to undershifting.(9, 27-29) The lack of an increasing trend may however also represent selective quitting. Many smokers in England quit during the study period, and smoking prevalence dropped from 19.3% in 2012 to 15.5% in 2016.(19) If smokers who spend more on tobacco are hit harder by a tax increase, they may be more likely to quit in response to increasing tax. The remaining smokers may therefore be those with lower levels of spending to begin with. The current study did not capture any effect of taxes on quitting.

We only collected data on expenditure on smoking, and not expenditure on alternative nicotine products. In England, e-cigarette use increased over the study period.(30) As a fifth of smokers use e-cigarettes,(31) we have underestimated smokers' expenditure on nicotine. Decreases in tobacco consumption may in part have been due to switching to dual-use of combustible cigarettes and e-cigarettes, and the inverse association found between cigarette consumption and smoking cost would have been somewhat weaker if expenditure on e-cigarettes would have been taken into account.

Interpretation

Our results are in line with previous findings that RYO cigarettes are much cheaper than factory-made cigarettes(9) as we found that switching from factory-made to RYO cigarettes is an effective cost-mitigating strategy. In our data, the proportion of RYO use increased over time. This increase has been observed since the early 2000's(29) and appears to continue over time.(8) Proportionally increased use is likely to be a response to an increasing gap in prices between factory-made and RYO.(9) Switching to RYO may have serious public health consequences, as smokers using RYO tobacco are much less sensitive to further price increases.(32) Moreover, smokers from lower social grades are more likely to use RYO tobacco(29), which makes the increasing price gap likely to contribute to growing socioeconomic inequalities in smoking.(12) In order to encourage smoking cessation across social grades, taxes on RYO tobacco need to increase to the same level as factory-made cigarettes, which may be achieved through continued larger increases in RYO tobacco taxes.(8, 17, 33)

We found a small increase in the proportion of smokers who reported purchasing tobacco from illicit or cross-border sources. This is in line with an overview by Rowell et al., showing that the most reliable information on illicit tobacco does not show dramatic increases in use.(34) An analysis of 2002-2014 UK ITC data showed no increases in use of self-reported sources outside the UK or from informal sellers.(8) Illicit trade in cigarettes, measured by the tax gap between consumption and sales of tobacco, decreased from 16% in 2005-2006 to 8% in 2014-2015.(22) However, between 2015-16 and 2016-17, the tax gap increased to 15%,(22) which this paper reflects. The increase in tax gap in recent years seems mainly due to a decline in consumption rather than a growth in illicit trade.(22) The tax gap for RYO tobacco strongly declined from 60% in 2005 to 28% in 2017.(22)

We found that, in line with previous findings,(8, 12, 18) individuals from lower social grades were less likely to purchase tobacco abroad and more likely to use illicit sources, than smokers from higher social grades. Although statistical power was limited, associations between consumption levels and costs tended to be stronger among individuals in the high social grade compared with the low social grade. This may be because smokers in lower social grades are more likely to use lower priced brands,(29) for which the reduction of consumption has a smaller effect on the total cost of smoking. The

For peer review only - http://bmjopen.bmj.com/site/about/guidelines.xhtml

association between illicit sources and the cost of smoking tended to be stronger in smokers from lower social grades, which may be explained by the frequency of using these sources, if smokers from lower social grades use illicit sources on a more regular basis.

Implications

In order to have actual costs of smoking increase above inflation, this study suggests that tax increases during the period of study were not enough to impact weekly tobacco expenditure. Changes in tobacco taxation policy are required, such as sudden larger tax increases, as called for elsewhere,(35) as these would be more impactful. However, tobacco taxation policies need to be designed in a way that takes industry strategies across brand segments and product types into account.(9, 27) As previously called for,(8, 9, 17, 27, 29, 33) this study makes a strong case for continued higher relative tax increases on RYO tobacco products compared with factory-made cigarettes. Other strategies may include maximising specific taxation, strong minimum price policies, plain packaging that removes price promotions from packs (already in effect in the UK), restricting brands to one variant and preventing the introduction of new brands.(9, 27, 36, 37)

Although illicit tobacco formed only a minor threat to the costliness of smoking, a continued increase is undesirable. Action at the national and international level, including effective implementation of the FCTC protocol to eliminate illicit trade, has potential to reduce illicit trade in the near future.(38-40)

Conclusions

At a time when tax increases were designed to raise the cost of tobacco 2-5% above inflation annually, weekly spend on tobacco by smokers in England did not change above inflation. Our study showed that smokers commonly reduce consumption and switch to hand-rolled tobacco, particularly those of more disadvantaged social grades, but that the use of illicit and cross-border sources of tobacco was much less common and hardly contributed to total expenditure on tobacco. Strong future tobacco taxation policies are needed, that take industry strategies across brand segments and product types into account.

For peer review only

For peer review only - http://bmjopen.bmj.com/site/about/guidelines.xhtml

Funding

This work was supported by the National Institute for Health Research Public Health Research (project number 13/43/58). The Smoking Toolkit Study is currently primarily funded by Cancer Research UK (C1417/A22962; C44576/A19501) and has previously also been funded by Pfizer, GSK, and the Department of Health. Department of Health Disclaimer: The views and opinions expressed therein are those of the authors and do not necessarily reflect those of the Public Health Research programme, NIHR, NHS or the Department of Health.

No funders had any involvement in the design of the study, the analysis or interpretation of the data, the writing of the report, or the decision to submit the paper for publication.

Competing interests

RW undertakes consultancy and research for and receives travel funds and hospitality from manufacturers of smoking cessation medications. EB and JB have received unrestricted research funding from Pfizer. MK, TP, AG, and AM have no interests to declare.

Author contributions

JB, AM and RW conceived and designed the study. MAGK prepared and analysed the data, interpreted the findings and drafted the manuscript. AM, TP, JB, RW, EB and AG contributed to the interpretation of the data and provided critical revisions to the manuscript. All authors approved the final version of the paper for submission.

Data sharing statement

For access to the Smoking Toolkit Study please contact Dr Jamie Brown, jamie.brown@ucl.ac.uk.

For peer review only

For peer review only - http://bmjopen.bmj.com/site/about/guidelines.xhtml

REFERENCES

1. World Health Organization. WHO report on the gobal tobacco epidemic, 2008: The MPOWER package. Geneva; 2008 2008.
2. Phlips L. Applied Consumption Analysis: Advanced Textbooks in Economics: Elsevier; 2014.
3. Chaloupka FJ, Yurekli A, Fong GT. Tobacco taxes as a tobacco control strategy. Tobacco Control. 2012;21(2):172-80.
4. Biener L, Aseltine Jr RH, Cohen B, Anderka M. Reactions of adult and teenaged smokers to the Massachusetts tobacco tax. American Journal of Public Health. 1998;88(9):1389-91.
5. Brown T, Platt S, Amos A. Equity impact of population-level interventions and policies to reduce smoking in adults: a systematic review. Drug and alcohol dependence. 2014;138:7-16.
6. Hiscock R, Bauld L, Amos A, Platt S. Smoking and socioeconomic status in England: the rise of the never smoker and the disadvantaged smoker. Journal of Public Health. 2012;34(3):390-6.
7. Bosdriesz JR, Willemsen MC, Stronks K, Kunst AE. Socioeconomic inequalities in smoking cessation in 11 European countries from 1987 to 2012. J Epidemiol Community Health. 2015:jech-2014-205171.
8. Partos TR, Gilmore AB, Hitchman SC, Hiscock R, Branston JR, McNeill A. Availability and use of cheap tobacco in the UK 2002-2014: Findings from the International Tobacco Control Project. Nicotine & Tobacco Research. 2017:ntx108.
9. Hiscock R, Branston JR, McNeill A, Hitchman SC, Partos TR, Gilmore AB. Tobacco industry strategies undermine government tax policy: evidence from commercial data. Tobacco Control. 2017:tobaccocontrol-2017-053891.
10. Choi K, Hennrikus D, Forster J, St Claire AW. Use of price-minimizing strategies by smokers and their effects on subsequent smoking behaviors. Nicotine & Tobacco Research. 2012;14(7):864-70.
11. Licht AS, Hyland AJ, O'Connor RJ, Chaloupka FJ, Borland R, Fong GT, et al. How do price minimizing behaviors impact smoking cessation? Findings from the International Tobacco Control (ITC) Four Country Survey. International journal of environmental research and public health. 2011;8(5):1671-91.
12. Licht AS, Hyland AJ, O'Connor RJ, Chaloupka FJ, Borland R, Fong GT, et al. Socio-economic variation in price minimizing behaviors: findings from the International Tobacco Control (ITC) Four Country Survey. International journal of environmental research and public health. 2011;8(1):234-52.
13. White VM, Gilpin EA, White MM, Pierce JP. How do smokers control their cigarette expenditures? Nicotine & tobacco research. 2005;7(4):625-35.
14. Xu X, Pesko MF, Tynan MA, Gerzoff RB, Malarcher AM, Pechacek TF. Cigarette price-minimization strategies by US smokers. American journal of preventive medicine. 2013;44(5):472-6.
15. Pesko MF, Licht AS, Kruger JM. Cigarette price minimization strategies in the United States: price reductions and responsiveness to excise taxes. nicotine & tobacco research. 2013;15(11):1858-66.
16. Pesko MF, Kruger J, Hyland A. Cigarette price minimization strategies used by adults. American journal of public health. 2012;102(9):e19-e21.
17. Young D, Wilson N, Borland R, Edwards R, Weerasekera D. Prevalence, correlates of, and reasons for using roll-your-own tobacco in a high RYO use country: findings from the ITC New Zealand survey. Nicotine Tob Res. 2010;12(11):1089-98.
18. Nagelhout GE, van den Putte B, Allwright S, Mons U, McNeill A, Guignard R, et al. Socioeconomic and country variations in cross-border cigarette purchasing as tobacco tax avoidance strategy. Findings from the ITC Europe Surveys. Tobacco control. 2013:tobaccocontrol-2012-050838.
19. Office for National Statistics. Dataset: Smoking habits in the UK and its constituent countries.; 2018.
20. World Health Organization. WHO report on the global tobacco epidemic 2015: raising taxes on tobacco: World Health Organization; 2015.

For peer review only - http://bmjopen.bmj.com/site/about/guidelines.xhtml

21. Action on Smoking and Health. Timeline of changes in tobacco taxation in the UK from 1993 to the present 2017 [cited 2017. Available from: http://ash.org.uk/information-and-resources/taxation-illicit-trade/taxation/ash-analysis-of-tobacco-tax-increases-in-the-united-kingdom/.
22. HM Revenue & Customs. Measuring tax gaps 2017 edition. Tobacco tax gap estimates for 2016-17.; 2017.
23. Fidler JA, Shahab L, West O, Jarvis MJ, McEwen A, Stapleton JA, et al. 'The smoking toolkit study': a national study of smoking and smoking cessation in England. BMC Public Health. 2011;11(1):1.
24. CPI INDEX 00: ALL ITEMS 2015=100, (2017).
25. Wood SN. Generalized additive models: an introduction with R: Chapman and Hall/CRC; 2017.
26. Guindon GE, Driezen P, Chaloupka FJ, Fong GT. Cigarette tax avoidance and evasion: findings from the International Tobacco Control Policy Evaluation Project. Tobacco control. 2013:tobaccocontrol-2013-051074.
27. Gilmore AB, Tavakoly B, Taylor G, Reed H. Understanding tobacco industry pricing strategy and whether it undermines tobacco tax policy: the example of the UK cigarette market. Addiction. 2013;108(7):1317-26.
28. Marsh L, Cameron C, Quigg R, Hoek J, Doscher C, McGee R, et al. The impact of an increase in excise tax on the retail price of tobacco in New Zealand. Tobacco control. 2015:tobaccocontrol-2015-052259.
29. Gilmore AB, Tavakoly B, Hiscock R, Taylor G. Smoking patterns in Great Britain: the rise of cheap cigarette brands and roll your own (RYO) tobacco. Journal of public health. 2015;37(1):78-88.
30. Beard E, Brown J, Michie S, West R. Is prevalence of e-cigarette and nicotine replacement therapy use among smokers associated with average cigarette consumption in England? A time-series analysis. BMJ open. 2018;8(6):e016046.
31. Brown J, West R, Beard E, Michie S, Shahab L, McNeill A. Prevalence and characteristics of e-cigarette users in Great Britain: findings from a general population survey of smokers. Addictive behaviors. 2014;39(6):1120-5.
32. Tait P, Rutherford P, Saunders C. Do consumers of manufactured cigarettes respond differently to price changes compared with their Roll-Your-Own counterparts? Evidence from New Zealand. Tobacco control. 2013:tobaccocontrol-2013-051010.
33. Young D, Yong H-H, Borland R, Shahab L, Hammond D, Cummings KM, et al. Trends in roll-your-own smoking: findings from the ITC Four-Country Survey (2002–2008). Journal of environmental and public health. 2012;2012.
34. Rowell A, Evans-Reeves K, Gilmore AB. Tobacco industry manipulation of data on and press coverage of the illicit tobacco trade in the UK. Tobacco control. 2014;23(e1):e35-e43.
35. Partos TR, Branston JR, Hiscock R, Gilmore AB, McNeill A. Individualised tobacco affordability in the UK 2002–2014: findings from the International Tobacco Control Policy Evaluation Project. Tobacco control. 2019:tobaccocontrol-2017-054027.
36. Chaloupka FJ. Maximizing the public health impact of alcohol and tobacco taxes. American journal of preventive medicine. 2013;44(5):561-2.
37. Golden SD, Smith MH, Feighery EC, Roeseler A, Rogers T, Ribisl KM. Beyond excise taxes: a systematic review of literature on non-tax policy approaches to raising tobacco product prices. Tobacco Control. 2016;25(4):377-85.
38. McNeill A, Iringe-Koko B, Bains M, Bauld L, Siggens G, Russell A. Countering the demand for, and supply of, illicit tobacco: an assessment of the 'North of England Tackling Illicit Tobacco for Better Health'Programme. Tobacco control. 2013:tobaccocontrol-2013-050957.
39. HM Revenue & Customs. Customs (2015). Tackling illicit tobacco: From leaf to light 2017 [
40. World Health Organization. Protocol to eliminate illicit trade in tobacco products. 2013.

For peer review only - http://bmjopen.bmj.com/site/about/guidelines.xhtml

FIGURES AND TABLES

Table 1: Weighted description of socio-demographics, cost-minimising strategies, and cost of smoking in the overall population of smokers and by social grade.

	Overall population	Social grade		
		**Low (N=7,032)**	**Middle (N=8,138)**	**High (N=1,797)**
Age distribution, % (95%CI)				
18 – 24	17.2 (16.6 to 17.8)	18.5 (17.6 to 19.5)	18.2 (17.3 to 19.1)	10.4 (9.0 to 11.9)
25 – 34	22.1 (21.4 to 22.8)	23.2 (22.1 to 24.4)	22.0 (21.0 to 23.0)	19.4 (17.5 to 21.6)
35 – 44	19.4 (18.7 to 20.1)	18.6 (17.6 to 19.6)	19.3 (18.3 to 20.2)	21.8 (19.7 to 24.0)
45 – 54	18.6 (18.0 to 19.3)	17.4 (16.4 to 18.4)	18.9 (18.0 to 19.9)	20.7 (18.7 to 22.8)
55 – 64	12.5 (12.0 to 13.1)	12.3 (11.5 to 13.1)	12.1 (11.4 to 12.9)	14.6 (13.0 to 16.4)
65+	10.2 (9.7 to 10.6)	10.0 (9.3 to 10.7)	9.5 (8.9 to 10.1)	13.1 (11.6 to 14.6)
Gender, % (95%CI)				
Male	47.2 (46.4 to 48.0)	52.1 (5038 to 53.3)	45.3 (44.2 to 46.5)	41.7 (39.3 to 44.2)
Female	52.8 (52.0 to 53.6)	47.9 (46.6 to 49.2)	54.7 (53.5 to 55.8)	58.3 (55.8 to 60.7)
Social grade, % (95%CI)				
Low	35.7 (34.9 to 36.5)	-	-	-
Middle	50.0 (49.1 to 50.8)	-	-	-
High	14.4 (13.7 to 15.0)	-	-	-
Factory-made cigarette consumption in cigarettes/week, mean (95%CI)	45.9 (45.0 to 46.8)	43.0 (41.6 to 44.4)	46.8 (45.5 to 48.1)	50.1 (47.4 to 52.8)
RYO cigarette consumption in cigarettes/week, mean (95%CI)	35.2 (34.4 to 36.1)	44.4 (42.9 to 45.9)	32.4 (31.2 to 33.5)	22.4 (20.3 to 24.5)
Use of illicit sources, % (95%CI)	8.1 (7.7 to 8.6)	10.0 (9.2 to 10.8)	7.7 (7.1 to 8.4)	5.1 (4.1 to 6.3)
Cross-border purchase, % (95%CI)	8.2 (7.7 to 8.7)	4.6 (4.1 to 5.2)	9.2 (8.5 to 9.9)	13.5 (11.9 to 15.3)
Cost of smoking in £/week, mean (95%CI)	23.3 (23.0 to 23.6)	22.8 (22.3 to 23.3)	23.5 (23.1 to 23.9)	23.9 (22.9 to 24.9)

Figure 1: Weighted trends in cost of smoking, cigarette consumption, use of illicit sources, and cross-border purchase among smokers in England.

For peer review only

For peer review only - http://bmjopen.bmj.com/site/about/guidelines.xhtml

Table 2: Weighted percentage difference in cost of smoking for socio-demographics and cost-minimising strategies from Generalised Additive Models (GAM).

	Percentage difference in cost of smoking (100β with 95%CI)		
	**Model 1**<br>Baseline model	**Models 2 to 5**<br>Adjusted for socio-demographics	**Model 6**<br>All variables
Age			
Per 10 years increase	7.21 (6.42 to 8.01)		0.14 (-0.42 to 0.70)
Gender			
Male	ref		ref
Female	2.22 (-0.34 to 4.78)		0.11 (-1.69 to 1.91)
Social grade			
Low	ref		ref
Middle	-1.08 (-3.88 to 1.72)		1.44 (-0.52 to 3.40)
High			-1.50 (-4.33 to 1.32)
Factory-made cigarette consumption[a]			
Per 10 cigarettes decrease		-10.17 (-10.35 to -9.99)	-12.99 (-13.18 to -12.80)
RYO cigarette consumption[b]			
Per 10 cigarettes decrease		1.19 (1.44 to 0.94)	-5.86 (-6.06 to -5.66)
Use of illicit sources[c]			
No use of illicit sources in last 6 months		ref	ref
Used illicit sources in last 6 months		-5.91 (-10.59 to -1.23)	-9.64 (-12.94 to -6.33)
Cross-border purchase[d]			
No cross-border purchase in last 6 months		ref	ref
Cross-border purchase in last 6 months		-7.04 (-11.72 to -2.37)	-9.47 (-12.74 to -6.20)

RYO = roll-your-own

[a] 50.4% of variance in spending accounted for by factory-made cigarette consumption.

[b] 9.5% of variance in spending accounted for by RYO cigarette consumption.

[c] 0.1% of variance in spending accounted for by use of illicit sources of tobacco.

[d] 0.1% of variance in spending accounted for by use of cross-border sources of tobacco.

For peer review only

Table 3: Weighted percentage difference in cost of smoking for socio-demographics and cost-minimising strategies from Generalised Additive Models (GAM), stratified by social grade.

	Social grade				
	Low (N=7,032)	Middle (N=8,138)		High (N=1,797)	
	100β with 95%CI	100β with 95%CI	P-value for interaction, middle vs low	100β with 95%CI	P-value for interaction, high vs low
Age					
Per 10 years increase	-0.23 (-1.04 to 0.59)	-0.18 (-0.65 to 1.00)		0.27 (-1.65 to 2.19)	
Gender					
Male	ref	ref		ref	
Female	0.54 (2.10 to 3.18)	0.17 (-2.44 to 2.78)		-1.55 (-7.56 to 4.46)	
Factory-made cigarette consumption					
Per 10 cigarettes decrease	-11.70 (-11.41 to -11.98)	-13.20 (-12.93 to -13.47)	<0.001	-15.36 (-14.74 to -15.98)	<0.001
RYO cigarette consumption					
Per 10 cigarettes decrease	-4.95 (-4.68 to -5.21)	-6.15 (-5.84 to -6.45)	0.007	-7.90 (-7.10 to -8.70)	0.004
Use of illicit sources					
No use of illicit sources in last 6 months	ref	ref		ref	
Used illicit sources in last 6 months	-12.25 (-16.72 to -7.78)	-7.11 (-12.02 to -2.21)	0.028	-5.63 (-19.09 to 7.84)	0.078
Cross-border purchase					
No cross-border purchase in last 6 months	ref	ref		ref	
Cross-border purchase in last 6 months	-13.01 (-19.03 to -6.72)	-9.99 (-14.46 to -5.53)	0.264	-7.24 (-15.80 to 1.33)	0.053

RYO = roll-your-own

Note: All models were adjusted for all variables in the table.

Table 4: Increase per year in the weighted % difference in cost of smoking for cost-minimising strategies from Generalised Additive Models (GAM)

	Increase in the % difference in cost of smoking for each consecutive year (95%CI)	P-value for interaction
Factory-made cigarette consumption · time	-0.12 (-0.23 to 0.00)	0.051
RYO cigarette consumption · time	0.00 (-0.12 to 0.13)	0.992
Illicit sources · time	0.93 (-1.44 to 3.31)	0.441
Cross-border purchase · time	-0.23 (-2.54 to 2.09)	0.848

RYO = roll-your-own tobacco

Note: Models were adjusted for age, gender, social grade, and other cost-minimising strategies in the table.

For peer review only - http://bmjopen.bmj.com/site/about/guidelines.xhtml

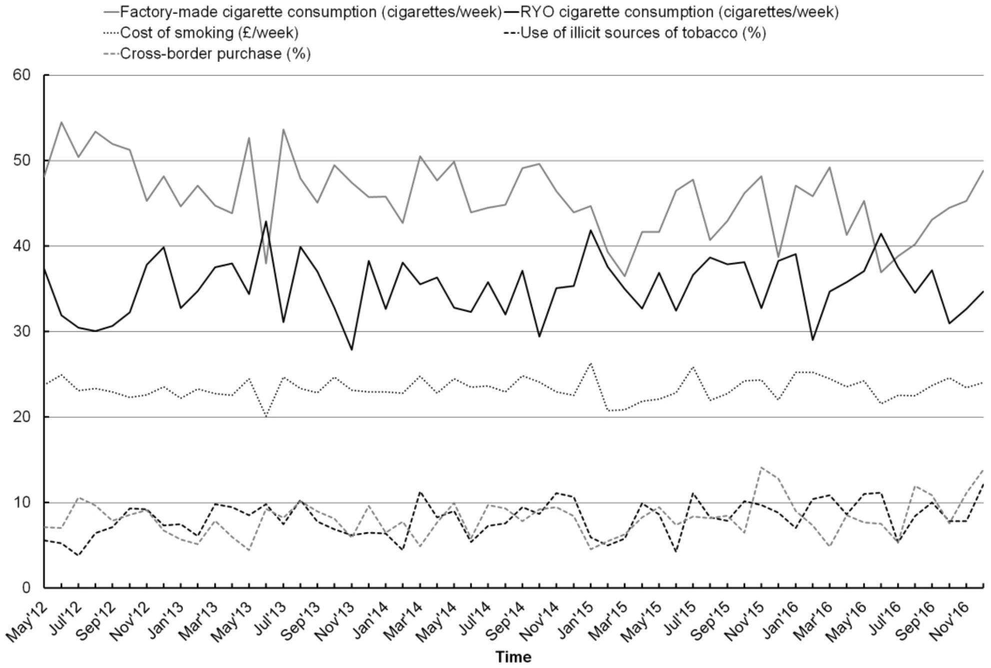

Figure 1: Weighted trends in cost of smoking, cigarette consumption, use of illicit sources, and cross-border purchase among smokers in England.

199x134mm (300 x 300 DPI)

SUPPLEMENTARY FILE

Mirte AG Kuipers, Timea R Partos, Ann McNeill, Emma Beard, Anna Gilmore, Robert West, Jamie Brown
Smokers' strategies across social grades to minimise the cost of smoking in a period with annual tax increases: Evidence from a national survey in England
BMJ Open

Supplementary Table 1: Results from weighted Generalised Additive Models (GAM) for cost of smoking, with total cigarette consumption and proportion of RYO use.

	**Percentage difference in cost of smoking (100β with 95%CI)**
	**Model 6**<br>Adjusted for other cost-minimising strategies
Age (per 10 years increase)	-0.29 (-0.85 to 0.27)
Gender	
Male	ref
Female	1.29 (-0.51 to 3.08)
Social grade	
Low	ref
Middle	2.35 (0.40 to 4.30)
High	-1.24 (-4.06 to 1.57)
Total cigarette consumption (per 10 cigarettes decrease)	-10.0 (-9.83 to -10.17)
Proportion of RYO cigarette consumption[a]	-70.41 (-72.29 to -68.53)
Use of illicit sources	-9.91 (-13.21 to -6.62)
Use of cross-border sources	-8.68 (-11.93 to -5.42)

[a] Represents the difference between 0% RYO and 100% RYO cigarettes of total consumption.

Supplementary Table 2: Results from weighted Generalised Additive Models (GAM) for price per cigarette.

	**Percentage difference in price per cigarette (100β with 95%CI)**
	**Model 6**<br>Adjusted for other cost-minimising strategies
Age (per 10 years increase)	-0.59 (-1.06 to -0.11)
Gender	
Male	ref
Female	1.70 (0.18 to 3.23)
Social grade	
Low	ref
Middle	5.41 (3.75 to 7.07)
High	9.52 (7.12 to 11.91)
Factory-made cigarette consumption (per 10 cigarettes decrease)	0.81 (0.97 to 0.65)
RYO cigarette consumption (per 10 cigarettes decrease)	7.76 (7.93 to 7.59)
Use of illicit sources	-12.10 (-14.90 to -9.29)
Use of cross-border sources	-11.17 (-13.94 to -8.40)

For peer review only

Supplementary Table 3: Results from weighted Generalised Additive Models (GAM) for cost of smoking, in the population from which respondents were not excluded based on the reported value for price/cigarette (N=17,789).

	**Percentage difference in cost of smoking (100β with 95%CI)**
	**Model 6**<br>Adjusted for other cost-minimising strategies
Age (per 10 years increase)	0.39 (-0.25 to 1.02)
Gender	
Male	ref
Female	1.48 (-0.57 to 3.52)
Social grade	
Low	ref
Middle	0.32 (-1.90 to 2.54)
High	-3.16 (-6.44 to 0.12)
Factory-made cigarette consumption[a]	
Per 10 cigarettes decrease	-12.27 (-12.48 to -12.05)
RYO cigarette consumption[b]	
Per 10 cigarettes decrease	-4.56 (-4.77 to -4.34)
Use of illicit sources[c]	
No use of illicit sources in last 6 months	ref
Used illicit sources in last 6 months	-9.12 (-12.83 to -5.42)
Cross-border purchase[d]	
No cross-border purchase in last 6 months	ref
Cross-border purchase in last 6 months	-14.87 (-18.57 to -11.18)

RYO = roll-your-own

For peer review only

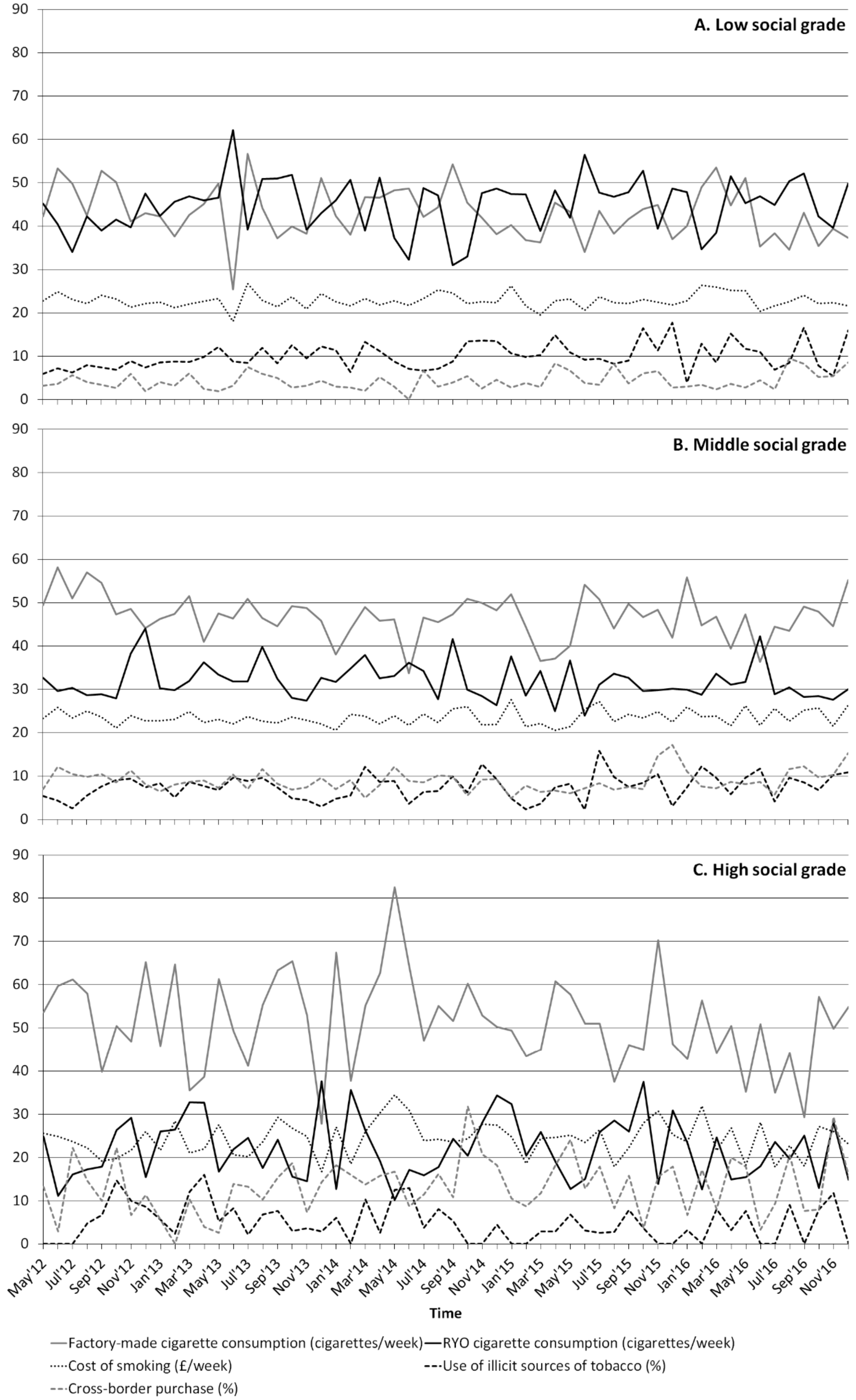

Supplementary Figure 1: Weighted trends in cost of smoking, cigarette consumption, use of illicit sources, and cross-border purchase among smokers in England, stratified by social grade.

STROBE Statement—Checklist of items that should be included in reports of ***cross-sectional studies***

	Page no.	Item No	Recommendation
**Title and abstract**	2	1	(*a*) Indicate the study's design with a commonly used term in the title or the abstract
	2		(*b*) Provide in the abstract an informative and balanced summary of what was done and what was found
	**Introduction**		
Background/rationale	3	2	Explain the scientific background and rationale for the investigation being reported
Objectives	3	3	State specific objectives, including any prespecified hypotheses
	**Methods**		
Study design	4	4	Present key elements of study design early in the paper
Setting	4	5	Describe the setting, locations, and relevant dates, including periods of recruitment, exposure, follow-up, and data collection
Participants	4	6	(*a*) Give the eligibility criteria, and the sources and methods of selection of participants
Variables	4,5	7	Clearly define all outcomes, exposures, predictors, potential confounders, and effect modifiers. Give diagnostic criteria, if applicable
Data sources/ measurement	4	8*	For each variable of interest, give sources of data and details of methods of assessment (measurement). Describe comparability of assessment methods if there is more than one group
Bias	5	9	Describe any efforts to address potential sources of bias
Study size	4	10	Explain how the study size was arrived at
Quantitative variables	4,5	11	Explain how quantitative variables were handled in the analyses. If applicable, describe which groupings were chosen and why
Statistical methods	5	12	(*a*) Describe all statistical methods, including those used to control for confounding
	5		(*b*) Describe any methods used to examine subgroups and interactions
	4		(*c*) Explain how missing data were addressed
	N/A		(*d*) If applicable, describe analytical methods taking account of sampling strategy
	5		(*e*) Describe any sensitivity analyses
	**Results**		
Participants	N/A	13*	(a) Report numbers of individuals at each stage of study—eg numbers potentially eligible, examined for eligibility, confirmed eligible, included in the study, completing follow-up, and analysed
	N/A		(b) Give reasons for non-participation at each stage
	N/A		(c) Consider use of a flow diagram
Descriptive data	5,12	14*	(a) Give characteristics of study participants (eg demographic, clinical, social) and information on exposures and potential confounders
	4		(b) Indicate number of participants with missing data for each variable of interest
Outcome data	fig1	15*	Report numbers of outcome events or summary measures
Main results	6,13	16	(*a*) Give unadjusted estimates and, if applicable, confounder-adjusted

For peer review only

			estimates and their precision (eg, 95% confidence interval). Make clear which confounders were adjusted for and why they were included
	N/A		(*b*) Report category boundaries when continuous variables were categorized
	N/A		(*c*) If relevant, consider translating estimates of relative risk into absolute risk for a meaningful time period
Other analyses	6, Suppl. tables	17	Report other analyses done—eg analyses of subgroups and interactions, and sensitivity analyses
**Discussion**			
Key results	6,7	18	Summarise key results with reference to study objectives
Limitations	7	19	Discuss limitations of the study, taking into account sources of potential bias or imprecision. Discuss both direction and magnitude of any potential bias
Interpretation	7,8	20	Give a cautious overall interpretation of results considering objectives, limitations, multiplicity of analyses, results from similar studies, and other relevant evidence
Generalisability	8	21	Discuss the generalisability (external validity) of the study results
**Other information**			
Funding	9	22	Give the source of funding and the role of the funders for the present study and, if applicable, for the original study on which the present article is based

*Give information separately for exposed and unexposed groups.

Note: An Explanation and Elaboration article discusses each checklist item and gives methodological background and published examples of transparent reporting. The STROBE checklist is best used in conjunction with this article (freely available on the Web sites of PLoS Medicine at http://www.plosmedicine.org/, Annals of Internal Medicine at http://www.annals.org/, and Epidemiology at http://www.epidem.com/). Information on the STROBE Initiative is available at www.strobe-statement.org.

For peer review only
